# Sumoylated SnoN interacts with HDAC1 and p300/CBP to regulate EMT-associated phenotypes in mammary organoids

**DOI:** 10.1038/s41419-023-05921-x

**Published:** 2023-07-07

**Authors:** Ayan Chanda, Anusi Sarkar, Lili Deng, Azad Bonni, Shirin Bonni

**Affiliations:** 1 Department of Biochemistry and Molecular Biology, Arnie Charbonneau Cancer Institute, Cumming School of Medicine, University of Calgary, Calgary, Canada; 2 Neuroscience and Rare Diseases, Roche Pharma Research and Early Development (pRED), Roche Innovation Center Basel, Basel, Switzerland

**Keywords:** Sumoylation, Breast cancer

## Abstract

Protein post-translational modification by the small ubiquitin-like modifier (SUMO) regulates the stability, subcellular localization, and interactions of protein substrates with consequences on cellular responses including epithelial-mesenchymal transition (EMT). Transforming growth factor beta (TGFβ) is a potent inducer of EMT with implications for cancer invasion and metastasis. The transcriptional coregulator SnoN suppresses TGFβ-induced EMT-associated responses in a sumoylation-dependent manner, but the underlying mechanisms have remained largely unknown. Here, we find that sumoylation promotes the interaction of SnoN with the epigenetic regulators histone deacetylase 1 (HDAC1) and histone acetylase p300 in epithelial cells. In gain and loss of function studies, HDAC1 suppresses, whereas p300 promotes, TGFβ-induced morphogenetic changes associated with EMT-related events in three-dimensional multicellular organoids derived from mammary epithelial cells or carcinomas. These findings suggest that sumoylated SnoN acts via the regulation of histone acetylation to modulate EMT-related effects in breast cell organoids. Our study may facilitate the discovery of new biomarkers and therapeutics in breast cancer and other epithelial cell-derived cancers.

## Introduction

Epithelial-mesenchymal transition (EMT) is a fundamental cellular process that is critical for multicellular organism development and homeostasis [1]. EMT also regulates cellular activity in diseased states including organ fibrosis and cancer [2]. During EMT, epithelial cells transdifferentiate to form less adherent and more motile mesenchymal cells via a complex cell-signaling network [3–5]. One of the major regulators of EMT is the transforming growth factor beta (TGFβ) signaling pathway [1, 6, 7]. Thus, identifying and characterizing novel regulators of TGFβ-induced EMT is a subject of intense interest in biology.

The canonical Smad pathway plays a major role in mediating TGFβ signaling from the cell surface to the nucleus. TGFβ triggers the assembly of an active transmembrane type I and II receptor ser/thr kinase complex that induces the phosphorylation of the Receptor-regulated Smads (R-Smad), Smad 2 and 3 [8, 9]. The phosphorylated Smad 2 and 3 (pSmad2/3) bind Smad4, the common partner Smad, and the complex accumulates in the nucleus, where it binds to specific DNA elements on TGFβ-target genes and in conjunction with diverse transcription factors and transcriptional coregulators controls the expression of a wide array of TGFβ-responsive gene in a cell- and context-specific manner that manifests as different responses including regulation of cell plasticity, e.g., EMT [10, 11].

The Ski-related novel protein N (SnoN) is a key component of the TGFβ signaling pathway [12–15]. SnoN can positively or negatively regulate TGFβ-signaling with consequences for biological responses [16, 17]. SnoN forms a complex with Smad2, Smad3, and Smad4 [18, 19]. Initial studies proposed a model whereby the closely related protein Ski leads to the dissociation of Smad2/3-Smad4 complex and thus suppresses TGFβ signaling [20]. In contrast, recent studies suggest that SnoN can form a ternary complex with and stabilizes the Smad2/3-Smad4 multiprotein complex [19]. SnoN associates with other proteins including chromatin remodelers such as histone deacetylases (HDACs) and histone acetyl transferases (HATs), which are recruited to promoters and enhancers of TGFβ responsive genes [15, 21]. How SnoN functions in modulating TGFβ signaling is regulated is an important question.

Sumoylation is a post-translational modification in which the protein small ubiquitin like modified (SUMO) is covalently conjugated to specific lysine residues in target proteins [22]. Sumoylation regulates target protein stability, localization and interaction with other proteins [22, 23]. Interestingly, SnoN is a SUMO target and Lysines 50 and 383, which reside within SUMO consensus motifs, are sites of sumoylation in SnoN. PIAS1 and TIF1γ represent two distinct SUMO E3 ligases that promote the sumoylation of SnoN [14, 24, 25]. Importantly, sumoylated SnoN mediates the ability of PIAS1 and TIF1γ to suppress TGFβ-induced EMT-related effects in epithelial cells and carcinoma-derived sheets and organoid cultures [24–26]. However, the downstream mechanisms by which sumoylated SnoN suppresses TGFβ-induced EMT had remained to be identified.

In this study, we find that sumoylation enhances SnoN association with the histone deacetylase HDAC1 and histone acetylase p300. Whereas HDAC1 suppresses, p300 promotes TGFβ-induced EMT-associated phenotypic changes in three-dimensional organoids derived from mammary epithelial cells or carcinomas. Our findings suggest that sumoylated SnoN suppresses EMT-associated responses by regulating histone acetylation in mammary cell-derived organoids.

## Methods

### Plasmids

To establish mammalian cells stably expressing SnoN, a pCaGip vector containing a cDNA to express a puromycin resistance marker was employed to generate constructs containing cDNA encoding wild type SnoN, a SUMO loss-of-function SnoNKdR, or a SUMO gain-of-function stable fusion SUMO-SnoN protein. The puromycin resistance marker and the SnoN proteins are encoded by a bicistronic transcript containing Internal Ribosomal Entry Site (IRES) as part of the pCaGip vector [25].

The pBJ5 vector containing SRα promoter, comprising the simian virus 40 early promoter and the R-U5 segment of human T-cell leukemia virus type 1 long terminal repeat, and cDNA encoding the C-terminally FLAG-tagged histone deacetylase 1 (HDAC1/FLAG) protein, is a gift from Wen-Ming Yang, Moffitt Cancer Center, USA [27]. CMV-based plasmid containing cDNA encoding the C-terminally hemagglutinin tagged p300 (p300/HA) protein, and a U6-based plasmid encoding an shRNA targeting the human and mouse p300 (p300i) are described [16].

Renilla Luciferase (RLuc) tagged-SnoN (Rluc/SnoN) expression vector is described [28]. SnoNKdR insert from the pCMV5B/MYC/SnoNKdR plasmid was subcloned using SalI and XbaI (NEB, Canada) digestion into SalI- and XbaI-digested pCMV5B/RLuc/SnoN backbone.

SUMO-SnoN insert was PCR-amplified from pCMV5B/SUMO-SnoN plasmid using the following primers: Forward Primer:5′ AGGCCTCGAGGATCTGACCAGGAGGCCAAAC 3′, and Reverse Primers:5′ CAAACTGGGATTTAAATGCAAAG 3′. The insert was digested using XhoI and SwaI (NEB, Canada) and subcloned into SalI- and SwaI-digested pCMV5B/RLuc/SnoN backbone to generate pCMV5B/Rluc/SUMO-SnoN.

The pBJ5/HDAC1/FLAG was used as a template to PCR amplify a fragment of HDAC1 flanked by SacII (NEB, Canada) and NdeI (NEB, Canada) restriction endonuclease cut sites. The following primers were used: Forward Primer:5′ CTAGAGCGGCCGCGGATCCGCCATGGCGAGAC 3’, and Reverse Primer:5′ GTCTCATATGTCCAGCACCGGGCAACGTTACGAA TGGTGT**G**ACCACCGCCTC 3′. The underlined and bold face nucleotide (G) in the reverse is to amplify HDAC1 in which Tyrosine 303 is converted to histidine (H) to generate a deacetylase inactive HDAC1 expression construct [29]. The PCR fragment was digested with and ligated with SacII and NdeI-digested pBJ5/HDAC1/FLAG vector to generate pBJ5/HDAC1YH/FLAG expressing vector.

HDAC1 RNA interference (HDAC1i) construct was generated in pU6/EGFP vector containing DNA encoding shRNAs against HDAC1 under the U6 promoter, and the green fluorescent protein (GFP) under CMV promoter. HDAC1i, targeting nucleotides 735–757 in exon 8 of human HDAC1, and corresponding regions in mouse and rat HDAC1, was generated using the following primer pair:

Forward Primer: 5′ GTCCAAAGTAATGGAGATGTTCCCAAGTTAACGGAACATCTCCATTACTTTGGACTTTTTG 3′ and Reverse Primer:5′ AATTCAAAAAGTCCAAAGTA ATGGAGATGTTCCGTTAACTTGGGAACATCTCCATTACTTTGGAC 3′.

All constructs were confirmed by sequencing.

### Cell lines, transfections, and reagents

The human embryonic kidney 293 T (HEK293T) epithelial cells, human MDA-MB-231 breast cancer cells, and NAMRU murine mammary gland (NMuMG) epithelial cells (source the American Type Culture Collection (ATCC), USA) were cultured in Dulbecco’s modified Eagle’s medium (DMEM) with high glucose and L-glutamine (Invitrogen, Canada) supplemented with 10% fetal bovine serum (FBS; Invitrogen, Canada). A total of 10 mg/ml recombinant human insulin (Invitrogen, Canada) was also added in the case of the NMuMG cells. In total 2 μg/ml puromycin was used in appropriate media to culture stable expression cell lines. Cells were maintained in a humidified incubator at 37 °C in 95% air and 5% CO_2_. All cell types were confirmed to be free of pathogenic Mycoplasma strains by a PCR-ELISA kit (Lonza, Switzerland).

HEK293T cells were transfected by the calcium-phosphate precipitation method [30]. The MDA-MB-231 and NMuMG cells were transfected using Lipofectamine 3000 reagent (Invitrogen, Canada) as per the manufacturer’s instructions [25, 26, 31].

Lyophilized recombinant human homodimerized mature transforming growth factor beta 1 (TGFβ1) (R&D systems, USA), referred to as TGFβ throughout the manuscript, was reconstituted in 30% acetonitrile and 0.01% trifluoroacetic acid at a stock concentration of 10 μM, divided into several 2 µL aliquots in 200 µL microfuge tubes, and stored at −80 °C. The TGFβ stock solution was then serially diluted to a final concentration of 100 pM in growth medium in 3D and 2D cell cultures. SB431542 (Millipore-Sigma, Canada), referred to as KI in this manuscript, is a small molecule selective inhibitor of the TGFβ type I receptor kinase, also known as ALK5, was reconstituted in dimethyl sulfoxide (DMSO) (Millipore-Sigma, Canada) as a 10 mM stock solution, which was used to obtain 10 µM final concentration in growth medium in organoid cultures as indicated.

### Immunoprecipitation and immunoblotting assays

Cells were seeded to achieve approximately a 30% confluency on the next day, when they were transfected with appropriate plasmids. Two days post-transfection, cells were lysed in TNTE (50 mM Tris, 150 mM NaCl, 0.5% [v/v] Triton X-100 and 1 mM EDTA) buffer-containing protease inhibitors and phosphatase inhibitors [25, 26, 31]. Cellular extracts were centrifuged at 14000 × *g* for 10 minutes at 4 °C. 95% of the supernatant was used for immunoprecipitation (IP) using appropriate antibodies and 5% was saved for protein determination and input sample analysis. The immunocomplexes and input lysates were subjected sodium dodecyl sulfate polyacrylamide gel electrophoresis (SDS-PAGE) to resolve the protein contents followed by transfer to nitrocellulose (NC) membranes [24, 25, 31]. The NC membranes were incubated with mouse anti-FLAG, mouse anti-HA, mouse anti-GFP (Santa Cruz, Canada), rabbit anti-SnoN (Proteintech, USA), rabbit anti-HDAC1 (Santa Cruz, Canada), mouse anti-p300 (Santa Cruz, Canada), rabbit anti-phospho-Smad2 (Abcam, USA), mouse anti-Smad2/3 (BD Transduction Laboratories, USA), or mouse anti-actin (Santa Cruz, Canada), as the primary antibody, and HRP-conjugated goat anti-mouse (Millipore Sigma, Canada) or donkey anti-rabbit IgG (VWR, Canada) as the secondary antibody. Immunocomplexes (80%) and input lysate (10-15%) from cells expressing RLuc alone or in fusion with SnoN were subjected to Renilla luciferase assays using the Renilla luciferase kit (Promega, USA) and the Orion II luminometer (Berthold Detection Systems, Germany) detection system. Prior to RLuc analysis, immunocomplexes were resuspended in TNE buffer-containing 0.1% Triton X-100 [28].

### Three-dimensional cultures

Three-dimensional cultures of cells were prepared in ultra-low attachment plates (8-well chamber slides (Millicell EZ Slide, Millipore) or 96-well flat-bottom, (BD Biosciences, ON, Canada)). Each well of the 8-well chamber slides and the 96-well dish were precoated with 75 and 50 μL, respectively, of 3 mg/mL Matrigel (BD Biosciences, Canada)-containing antibiotic-antimycotic-growth medium and incubated for 1 h in a tissue culture hood at 37 °C and 5% CO_2_ to allow formation of the Matrigel bed/cushion. Next, 75 μL or 50 μL of isolated cells resuspensions in 5 mg/ml Matrigel in growth medium, at a concentration of 10 cells/μL (NMuMG cells) and 6 cells/μL (MDA-MB-231 cells), were layered on top of the Matrigel cushion in each well of the 8-well chamber slide or 96-well dish, respectively, and incubated for 1 h at 37 °C and 5% CO_2_ to allow the upper Matrigel-cell layer to solidify. A total of 50 μL of complete medium was then carefully added to the Matrigel-cell layer. The 3D-cell cultures received specific treatments on the next and every third day for a total of 8 days of culturing. Differential interference contrast (DIC) images of six representative organoids from each well were captured using a microscope (Olympus IX70) using a 30× objective, after overall estimation and phenotype assessment of the total number of organoids per well (acini vs. filled for NMuMG organoids, non-deformed/spherical vs. deformed for NMuMG and MDA-MB-231 organoids) [24, 26, 31–34]. NMuMG and MDA-MB-231 cell-derived multicellular structures were assessed for hollow centered-(30–40 µm in diameter) and filled- spheres appearances, respectively. In addition, multicellular structures with shapes deviating from the outer round-smooth-surfaced phenotype, predominantly observed in vector-control cells, were quantified, and referred to as “deformed”. Deformed phenotypes included organoids showing invagination/budding and/or basal-surface single cell invasiveness into the matrix. In the case of NMuMG cell-derived multicellular structures, deformed multicellular structures also included filled-lumen organoids with greater than 100 µm diameter. Each experiment was repeated at least three independent times for statistical inference.

### Immunofluorescence and fluorescence cell-based analyses

The 8-day old 3D-organoid cultures were fixed with 4% formaldehyde and permeabilized using 0.5% cold Triton X-100 solution. The fixed-organoid cultures were blocked using 10% BSA in PBS and subjected to indirect immunofluorescence staining using an E-cadherin antibody (Cell Signaling Technology, Canada) as the primary antibody and goat anti-rabbit antibody conjugated to Cy5 dye (Jackson Laboratories, Canada) as the secondary antibody. The organoids also were incubated with the filamentous (F) actin stain tetramethyl-rhodamine isothiocyanate (TRITC)-conjugated phalloidin (Millipore-Sigma, Canada) and the DNA fluorescent dye Hoechst 33342 (Invitrogen, Canada) to visualize the actin cytoskeleton and nuclei, respectively, of cells. The slides were mounted, and fluorescence images of the multicellular colonies were captured using a fluorescence microscope with a 40× objective lens (Olympus Fluoview FV1000 microscope, Canada). Exposure times for E-cadherin, F-actin, and Hoechst-specific signals were kept constant in each experiment [24, 25, 32, 34]. Images of 2 to 3 organoids per experimental condition were captured out of more than six qualitatively-assessed organoids. TRITC-phalloidin stained multicellular structures were quantified for cortical actin organization and presented graphically.

### Migration-Scratch assay

A scratch assay was used to assess the migratory abilities of the MDA-MB-231 breast cancer cells under different conditions. To achieve this 0.5 ml 10% FBS-growth medium containing 5 × 10^5^ cells was added per well of a 12-well tissue culture plate that was kept in a 5% CO_2_ humidified incubator at 37 °C incubator for 24 h to allow cell monolayers reach near confluency. Confluent MDA-MB-231 cell monolayers were then washed and incubated at 37 °C in 0.2% FBS-containing DMEM medium overnight (serum starvation). Cells were removed along the midline of the serum-starved cell monolayers in each well using a 200 μL pipette tip (scratch), followed by a PBS wash to remove floating cells, and incubating the remaining monolayers under serum starvation, without or with 100 pM final concentration of TGFβ, for 30 h in a 5% CO_2_ humidified incubator at 37 °C. Cells and enclosing scratch were imaged at 4× objective of a DIC microscope (Olympus IX70) coupled to a digital camera at time 0 h and 30 h after initiating the scratch. To assess scratch width and closure, three images were obtained along the vertical axis of each scratch per well, and the width of the scratch (empty space) at three distinct positions per image were measured using ImageJ (National Institutes of Health, USA), averaged for the nine measurements per well. Scratch closure per well, i.e., experimental condition, is determined by subtracting the average width at 30 h from that at 0 h and expressing the difference relative to average width at 0 h.

### Statistical analyses

Replicates are used to account for intra-experimental variations in measurements of a particular parameter, which may otherwise lead to erroneous hypothesis testing and parameter estimation. Biological replicates incorporate the use of biologically distinct samples or cell culture populations in a specific type of experiment and are used to alleviate random biological variations which may be a source of noise or be part of the subject of study [35]. A minimum of three biological replicates were used for each type of experiment in studies described in this manuscript, except where stated, to facilitate statistical inference. Unpaired Student’s t-test (for two groups) or One-way analysis of variance (ANOVA) followed by Tukey-Kramer post-test (for more than two groups) using InStat (GraphPad InStat, San Diego, CA, USA) were performed to evaluate the statistical significance of data from biological replicates per experiment where appropriate. Values of *P* ≤ 0.05 were considered statistically significant. Data are presented graphically as mean ± standard error of the mean (SEM) for experiments with a minimum of three biological replicates.

## Results

### Sumoylated SnoN suppresses TGFβ-induced EMT in mammary epithelial organoids

**Fig. 1:**
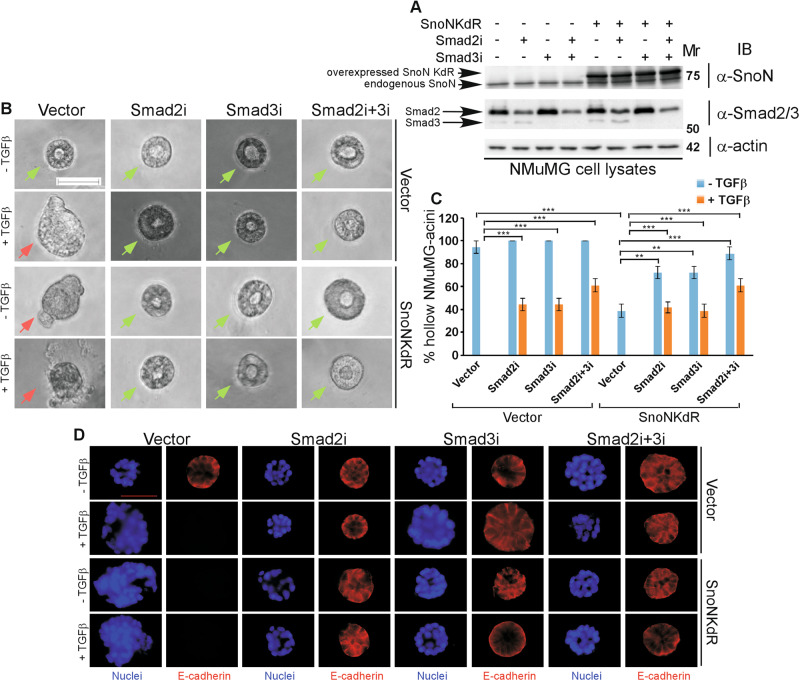
TGFβ-Smad2/3 pathway mediates the ability of the non-sumoylated SnoN to induce EMT in mammary epithelial organoids. **A** Smad2/3, SnoN, and actin (loading control) immunoblotting (IB) of lysates of NMuMG cells transfected with a stable vector control (−) or a plasmid stably expressing the SUMO loss of function SnoNKdR (+), with each transiently transfected with a control RNAi plasmid (−), or one expressing a Smad2-targetting shRNA Smad2i (+), a Smad3-targetting shRNA Smad3i (+), alone or together. **B** Representative differential interference contrast (DIC) light microscopy micrographs of live untreated (−) or 100 pM TGFβ-treated (+) 8-day old organoids of NMuMG cells transfected and assessed as in **A**. Green and red arrows indicate hollow and filled acinar organoids, respectively. **C** Bar graph depicts mean ± SEM proportion of hollow centred-acinar organoids expressed as a percentage of total colonies scored for each experimental condition from three biological replicates including the one with representative DIC images shown in **B**. **D** Representative fluorescence microscopy scans of E-cadherin-(immunocytochemistry-red) and nuclei-(Hoechst-blue) stained fixed 8-day old organoids of NMuMG cells transfected and assessed as in **A** to **C**. Mr indicates Markers’ molecular size. Statistical difference, ANOVA: **P ≤ 0.01, ***P ≤ 0.001. Scale bar indicates 50 μm.

The activated TGFβ-receptor complex induces the C-terminal phosphorylation of Smad2 and Smad3, which in turn transduce the signal from the cytoplasm to the nucleus leading to regulation of gene expression programs including those that promote EMT [9–11]. SnoN regulates TGFβ signaling by interacting with Smad2/3 raising the question of whether sumoylation of SnoN regulates the ability of Smad proteins to promote EMT. We first determined the role of SnoN in Smad-mediated EMT in non-transformed NMuMG mammary epithelial cells, because these cells represent a good model for studies of EMT [14, 24, 25, 32]. NMuMG cells cultured in the context of extracellular matrix (ECM) lead to organoids with a hollow lumen or acini. EMT-related changes manifest as morphological changes including lumen filling and/or deformation, loss or mislocalization of the epithelial marker E-cadherin, and loss or reduction in the cortical actin phenotype to a more diffused actin cytoskeleton (Fig. 1B–D, S1A–C) [24, 25, 33]. The protein SnoNKdR is a sumoylation-defective SnoN as the SUMO acceptor Lysine residues 50 and 383 are converted to arginine, which lacks the ability to be conjugated by SUMO [14, 24–26]. As expected, expression of SnoNKdR (Fig. 1A) promoted EMT-related changes including filling, deformation, E-cadherin loss/mislocalization, and cortical actin loss/reduction in the NMuMG organoid acini even in the absence of exogenous TGFβ (Fig. 1B–D, S1A–C) [24, 25]. In addition, small hairpin (sh) RNA-knockdown of endogenous Smad2 (Smad2i), Smad3 (Smad3i), alone or together (Fig. 1A) suppressed these TGFβ-EMT-like effects in three-dimensional NMuMG cell-derived organoids [32]. Importantly, knockdown of endogenous Smad2/3 led to significant suppression of SnoNKdR-induced EMT-related responses in NMuMG organoids (Fig. 1A–D, S1A–C).

**Fig. 2:**
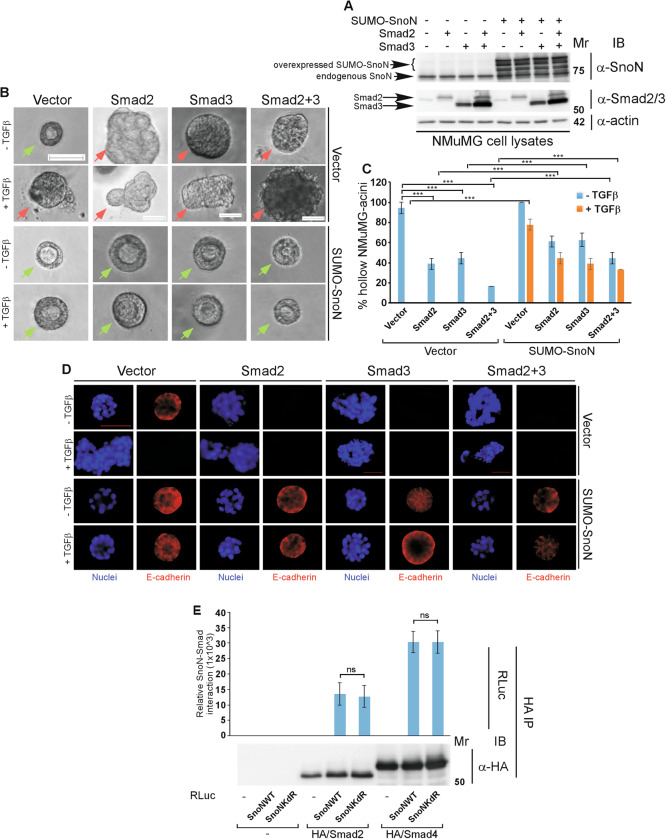
Sumoylation is important for SnoN to suppress Smad2/3-induced EMT but not SnoN-Smad association. **A**–**D** SUMO gain of function SnoN antagonizes EMT induction by expressed Smad2/3. **A** Smad2/3, SnoN, and actin (loading control) immunoblotting (IB) of lysates of NMuMG cells transfected with a stable vector control (−), or a plasmid stably expressing the SUMO gain of function SnoN SUMO-SnoN (+), with each transiently transfected with a vector control (−), or a plasmid expressing Smad2 (+) or Smad3 (+), alone or together. **B** Representative differential interference contrast (DIC) light microscopy micrographs of live untreated (−) or 100 pM TGFβ-treated (+) 8-day old organoids of NMuMG cells transfected and assessed as in **A**. Green and red arrows indicate hollow or filled acinar organoids, respectively. **C** Bar graph depicts mean ± SEM proportion of hollow acinar organoids expressed as a percentage of total colonies counted for each experimental condition from three biological replicates including the replicate with representative DIC images shown in **B**. **D** Representative fluorescence microscopy scans of E-cadherin-(immunocytochemistry-red) and nuclei-(Hoechst-blue) stained fixed 8-day old organoids of NMuMG cells transfected and assessed as in **A** to **C**. **E** *Sumoylation does not impact SnoN-Smad interaction*. Lysates of HEK293T cells transfected with a plasmid encoding only the protein Renilla luciferase (RLuc) (−), or in fusion with the wild type SnoN (SnoNWT), or the SUMO loss of function SnoNKdR, together with a vector control (−) alone, or a plasmid encoding an hemagglutinin-tagged Smad2 (HA/Smad2) or Smad4 (HA/Smad4), and a plasmid encoding a constitutively active TGFβ type I receptor kinase in which Threonine 204 is converted to aspartate (TβRI TD), were subjected to Smad2/Smad4 immunoprecipitation (α-HA IP), followed by Renilla luciferase assay (RLuc) or Smad immunoblotting (α-HA IB) of 80% and 20%, respectively, of the Smad immunocomplexes. Graphical data show mean ± SEM (three biological replicates) of luciferase activity coimmunoprecipitated by Smad2/4 expressed relative to its respective input luciferase activity. Mr indicates Markers’ molecular size. Statistical difference, ANOVA: ***P ≤ 0.001, ns not significant. Scale bar indicates 50 μm.

The SUMO-SnoN fusion acts as SUMO gain of function SnoN including in EMT [24–26]. Accordingly, SUMO-SnoN (Fig. 2A) suppressed exogenous TGFβ-induced filling and/or disorganization, loss/mislocalization of E-cadherin, and loss/reduction of cortical actin organization in the 3D-NMuMG cell-derived organoids (Fig. 2B–D, S2A–C) [25]. Importantly, expression of SUMO-SnoN suppressed the ability of overexpressed Smad2 and Smad3 (Fig. 2A) to induce EMT-like responses in the non-transformed mammary epithelial cell-derived organoids (Fig. 2B–D, S2A–C). Collectively, these data suggest that SnoN suppresses TGFβ-induced EMT-related effects in mammary epithelial cells in a Smad-dependent manner.

We asked whether sumoylation affects the ability of SnoN to interact with Smad proteins. SnoN interacts with Smad2/3 and Smad4 via distinct regions in SnoN [36, 37]. SnoN interacted with Smad2 and Smad4 in the presence of constitutively activated TGFβ receptor I (TβRI TD) (Fig. 2E, S2D) [15, 38, 39]. However, both SnoNKdR and wild type SnoN associated effectively with Smad2 and Smad4. These data suggest that sumoylation has little or no effect on the association of SnoN with Smad proteins.

### Sumoylation regulates the ability of SnoN to associate with histone acetylation modulators with functional significance for EMT induction in epithelial cell-derived organoids

**Fig. 3:**
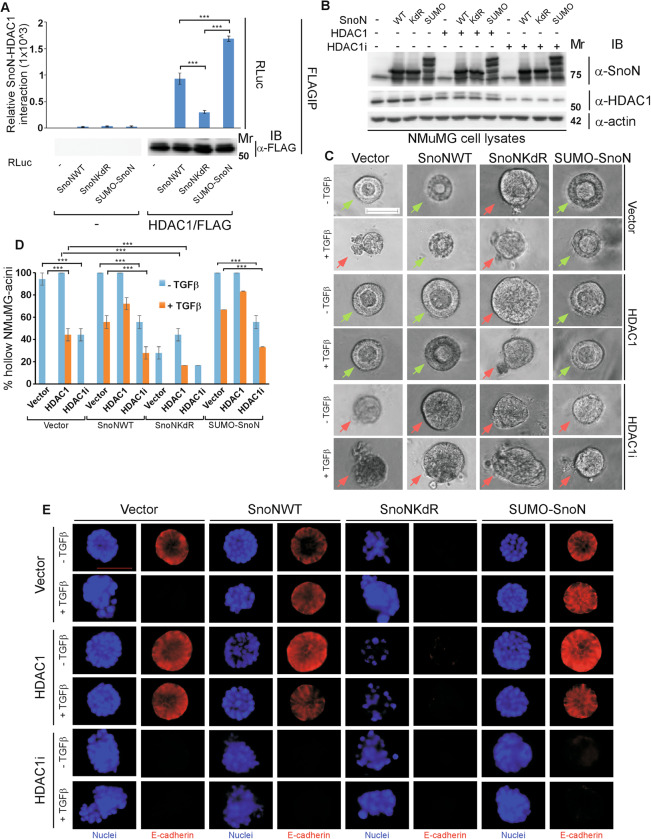
Sumoylation promotes SnoN interaction with HDAC1, to suppress TGFβ-induced EMT. **A** Sumoylation enhances SnoN-HDAC1 association. Lysates of HEK293T cells transfected with a plasmid encoding only the protein Renilla luciferase (RLuc) (−), or in fusion with the wild type SnoN, the SUMO loss of function SnoNKdR, or the SUMO gain of function SUMO-SnoN, together with a control vector (−), or a plasmid encoding a C-terminally FLAG-tagged HDAC1 (HDAC1/FLAG), were subjected to HDAC1 immunoprecipitation (α-FLAG IP), followed by Renilla luciferase assay (RLuc) or HDAC1 immunoblotting (α-FLAG IB) of 80% and 20%, respectively, of the HDAC1 immunocomplexes. Graphical data show mean ± SEM (three biological replicates) of luciferase activity coimmunoprecipitated by HDAC1 expressed relative to its respective input luciferase activity. **B**–**E** Sumoylated SnoN acts via HDAC1 to suppress EMT in mammary epithelial organoids. **B** SnoN, HDAC1 and actin (loading control) immunoblotting (IB) of lysates of NMuMG cells transfected with a stable vector control (−), or a plasmid stably expressing the wild type SnoN (WT), the SUMO loss of function SnoNKdR (KdR), or the SUMO gain of function SUMO-SnoN (SUMO), with each transiently transfected with vector controls (−), or a plasmid expressing the protein HDAC1 (+), or one encoding the HDAC1-targetting shRNA HDAC1i (+). **C** Representative differential interference contrast (DIC) light microscopy micrographs of live untreated (−) or 100 pM TGFβ-treated (+) 8-day old organoids of NMuMG cells transfected and assessed as in B. Green and red arrows indicate hollow and filled/disorganized acinar organoids, respectively. **D** Bar graph depicts mean ± SEM of proportion of hollow acinar organoids expressed as a percentage of total colonies scored for each experimental condition from three biological replicates including the replicate with representative DIC images shown in **C**. **E** Representative fluorescence microscopy scans of E-cadherin-(immunocytochemistry-red) and nuclei-(Hoechst-blue) stained of fixed 8-day old organoids of NMuMG cells transfected and assessed as in **B**–**D**. Mr indicates Markers’ molecular size. Statistical difference, ANOVA: ***P ≤ 0.001. Scale bar indicates 50 μm.

SnoN interacts with HDAC1 and thereby inhibits Smad-dependent transcription [15, 21]. We asked whether sumoylation affects the association of SnoN with HDAC1. To address this question, we assessed the ability of the SUMO loss of function SnoNKdR and SUMO gain of function SUMO-SnoN, to associate with HDAC1 using Renilla luciferase-based coimmunoprecipitation assays. We confirmed that SnoN interacts with HDAC1 (Fig. 3A, S3A). We found that SnoNKdR interacted less effectively, and SUMO-SnoN interacted more effectively, as compared to wild type SnoN, with HDAC1 (Fig. 3A). Thus, these data suggest that sumoylation promotes the association of SnoN with HDAC1.

We next asked the functional consequences of the effect of sumoylation on SnoN-HDAC1 interaction. We first characterized the role of HDAC1 in the morphogenesis of mammary epithelial cell-derived organoids and in relationship to TGFβ-induced EMT. Increasing the protein abundance of HDAC1, even modestly (Fig. 3B), in cells suppressed exogenous TGFβ-induced EMT, as indicated by presence of hollow center, decreased deformation, maintenance of protein abundance and junctional localization of E-cadherin, and of cortical actin phenotypes of the NMuMG cell-derived acini (Fig. 3C–E, S3B–D). Conversely, RNAi-induced (HDAC1i) knockdown of endogenous HDAC1 (Fig. 3B) promoted an EMT-like phenotype in the 3D-NMuMG cell-derived acini, even in the absence of exogenous TGFβ (Fig. 3C–E, S3B–D). Expression of the sumoylation-defective SnoNKdR reduced EMT suppression by overexpressed HDAC1, whereas knockdown of endogenous HDAC1 abrogated the ability of SnoN or SUMO-SnoN to suppress EMT-like behaviour of the three-dimensional mammary epithelial organoids (Fig. 3B–E, S3B–D). Together, these data suggest that endogenous HDAC1 suppresses TGFβ-Smad-induced EMT-like effects in epithelial cell-derived organoids in a manner dependent on the sumoylation status of SnoN.

**Fig. 4:**
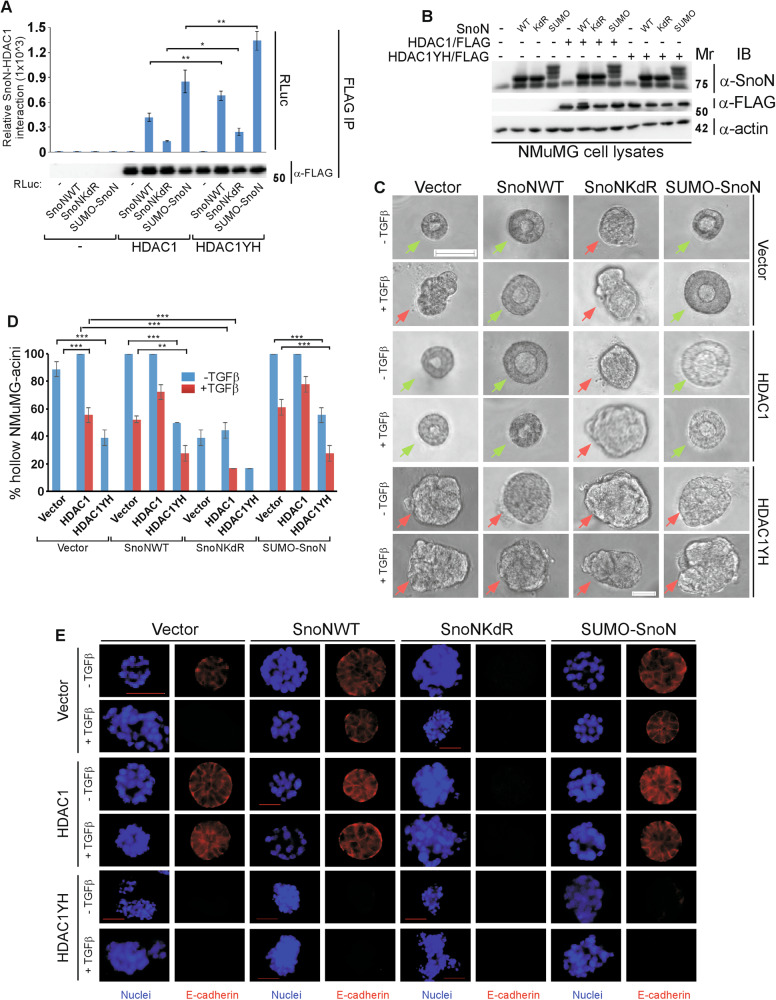
Sumoylated SnoN suppresses, in an HDAC1 deacetylase activity-dependent manner, TGFβ-induced EMT of mammary epithelial organoids. **A** Effect of HDAC1 acetylase activity on association with SnoN. Lysates of HEK293T cells transfected with a plasmid encoding only the protein Renilla luciferase (RLuc) (−), or in fusion with the wild type SnoN, the SUMO loss of function SnoNKdR, or the SUMO gain of function SUMO-SnoN, together with a control vector (−), or a plasmid encoding HDAC1/FLAG or the deacetylase inactive HDAC1YH/FLAG, were subjected to HDAC1 immunoprecipitation (α-FLAG IP), followed by Renilla luciferase assay (RLuc) or HDAC1 immunoblotting (α-FLAG IB) of 80% and 20%, respectively, of the HDAC1 immunocomplexes. Graphical data show mean ± SEM (three biological replicates) of luciferase activity coimmunoprecipitated by HDAC1 expressed relative to its respective input luciferase activity. *B-E) DAC1 acts in a deacetylase-dependent manner to mediate sumoylated SnoN-HDAC1 suppression of EMT*. **B** SnoN, HDAC1 and actin (loading control) immunoblotting (IB) of lysates of NMuMG cells transfected with a stable vector control (−), or a plasmid stably expressing the wild type SnoN (WT), the SUMO loss of function SnoNKdR (KdR), or the SUMO gain of function SUMO-SnoN (SUMO), with each transfected transiently with a vector control (−), or a plasmid encoding HDAC1 (+) or the deacetylase inactive HDAC1YH ( + ). **C** Representative differential interference contrast (DIC) light microscopy micrographs of untreated (−) or 100 pM TGFβ-treated (+) 8-day old organoids of NMuMG cells transfected and assessed as in **B**. Green and red arrows indicate acinar and filled organoids, respectively. **D** Bar graph depicts mean ± SEM proportion of acinar organoids expressed as a percentage of total colonies counted for each experimental condition from three biological replicates including the replicate with the representative DIC images shown in **C**. **E** Representative fluorescence microscopy scans of E-cadherin-(immunocytochemistry-red) and nuclei-(Hoechst-blue) stained of fixed 8-day old organoids of NMuMG cells transfected and assessed as in **B**–**D**. Mr indicates Markers’ molecular size. Statistical difference, ANOVA: *P ≤ 0.05, **P ≤ 0.01, ***P ≤ 0.001. Scale bar indicates 50 μm.

Next, we determined the role of HDAC1 deacetylase activity in mediating SnoN function in EMT. We first generated a deacetylase inactive HDAC1 mutant (HDAC1YH), in which the Tyrosine 303 residue was mutated to histidine [29]. SnoN associated more robustly with the deacetylase-dead HDAC1YH as compared to the native HDAC1 (Fig. 4A, S4A). Importantly, the enzymatically inactive HDAC1YH (Fig. 4B) phenocopied HDAC1 knockdown in promoting EMT-associated changes in the NMuMG cell-derived organoids even in the absence of exogenous TGFβ (Fig. 4C–E, S4B–D). Similarly, HDAC1YH decreased the ability of SnoN or SUMO-SnoN to suppress EMT induction by exogenous TGFβ (Fig. 4C–E, S4B–D). These data suggest that HDAC1 acts in deacetylase-dependent manner to mediate SnoN suppression of exogenous TGFβ-induced EMT-associated phenotypes in mammary epithelial cell-derived organoids (Fig. 4B–E, S4B–D). In other analyses, we found that induction of EMT upon knockdown of endogenous HDAC1 or increased expression of the deacetylase-inactive HDAC1YH (Fig. S5A–B) was reversed by incubation of the NMuMG organoid cultures with a TGFβ type I receptor kinase ((TβRI) inhibitor (KI) (Fig. S5C–E). These data suggest that endogenous HDAC1 acts in a deacetylase-dependent manner to suppresses the ability of endogenous/basal TGFβ-Smad signaling to induce EMT-related plastic effects in epithelial cell-derived organoids (Fig. S5C–E). Collectively, these findings suggest that sumoylation enhances the SnoN-HDAC1 association, which in turn suppresses, in an HDAC1 deacetylase-dependent manner, TGFβ-induced EMT in epithelial cell-derived organoids.

### Sumoylated SnoN suppression of TGFβ-induced EMT involves regulation of function of acetyl transferase p300

**Fig. 5:**
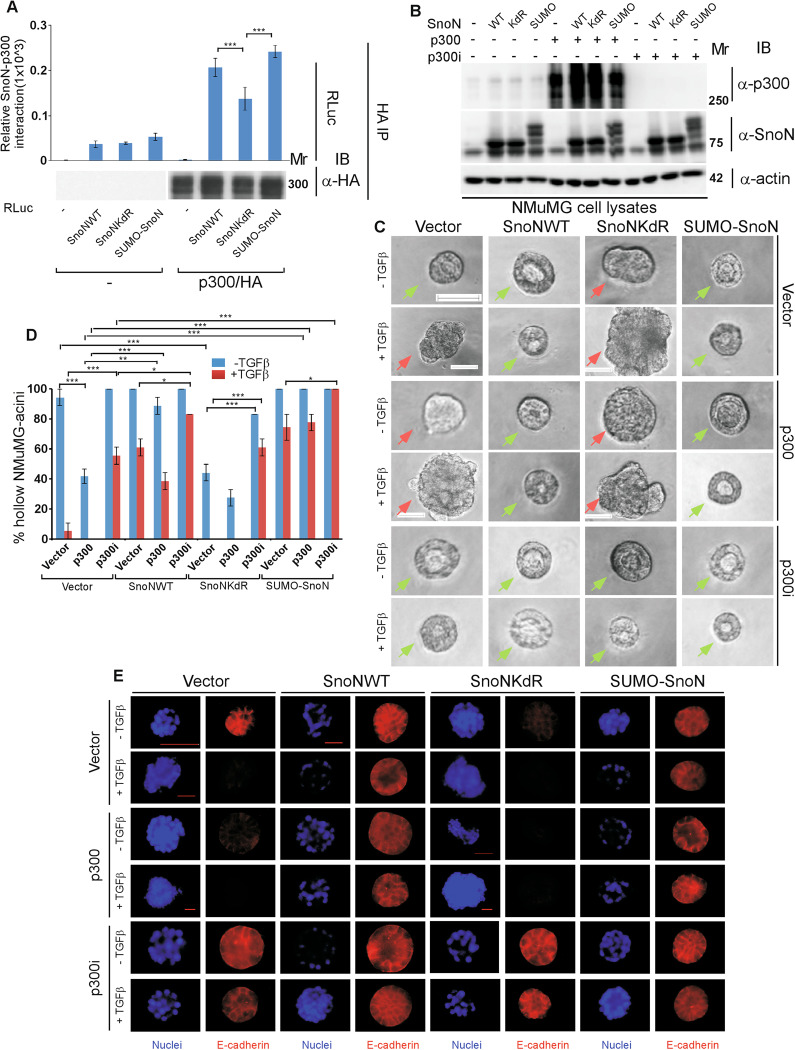
Sumoylation promotes SnoN-p300 interaction to suppress TGFβ-induced EMT in mammary epithelial organoids. **A** Sumoylation promotes SnoN-p300 association. Lysates of HEK293T cells transfected with a plasmid encoding only the protein Renilla luciferase (RLuc) (−), or in fusion with the wild type SnoN, the SUMO loss of function SnoNKdR, or the SUMO gain of function SUMO-SnoN, together with a control vector (−), or a plasmid expressing a C-terminally HA-tagged p300 (p300/HA), were subjected to p300 immunoprecipitation (α-HA IP), followed by Renilla luciferase assay (RLuc) or p300 immunoblotting (α-HA IB) of 80% and 20%, respectively, of the p300 immunocomplexes. Graphical data show mean ± SEM (three biological replicates) of luciferase activity coimmunoprecipitated by p300, expressed relative to its respective input luciferase activity. **B–E** *Sumoylated SnoN suppresses p300-induced EMT in mammary epithelial organoids*. **B** SnoN, p300 and actin (loading control) immunoblotting (IB) of lysates of NMuMG cells transfected with a stable vector control (−), or a plasmid stably expressing the wild type SnoN (WT), the SUMO loss of function SnoNKdR (KdR), or the SUMO gain of function SUMO-SnoN (SUMO), with each transfected transiently with vector controls (−), or a plasmid encoding the protein p300/HA (+), or the p300-targetting shRNA p300i (+). **C** Representative differential interference contrast (DIC) light microscopy micrographs of untreated (−) or 100 pM TGFβ-treated (+) 8-day old organoids of NMuMG cells transfected and assessed as in **B**. Green and red arrows indicate acinar and filled organoids, respectively. **D** Bar graph depicts mean ± SEM proportion of acinar organoids expressed as a percentage of total colonies counted for each experimental condition from three biological replicates including the replicate with representative DIC images shown in **C**. **E** Representative fluorescence microscopy scans of E-cadherin-(immunocytochemistry-red) and nuclei-(Hoechst-blue) stained of fixed 8-day old organoids of NMuMG cells transfected and assessed as in **B**–**D**. Mr indicates Markers’ molecular size. Statistical difference, ANOVA: *P ≤ 0.05, ***P ≤ 0.001. Scale bar indicates 50 μm.

The histone acetyltransferase p300 that promotes histone acetylation and regulates chromatin compactness, interacts with SnoN and thereby regulates gene expression regulation [16, 40]. We, therefore, determined if sumoylation of SnoN modulates the SnoN-p300 association. Luciferase-based coimmunoprecipitation analysis confirmed that SnoN and the SUMO gain of function SUMO-SnoN associate similarly with p300, whereas the SUMO loss of function SnoNKdR showed approximately 40% reduction in its ability to interact with p300 (Fig. 5A and S6A). These data suggest that sumoylation promotes SnoN-p300 complex formation (Fig. 5A).

That sumoylation enhances SnoN association with p300 raised the question of functional implication of this modification-enhanced interaction. To address this question, we focused on TGFβ-induced EMT. We found that p300 expression promoted, whereas knockdown of p300 (Fig. 5B) suppressed exogenous TGFβ-induced EMT-like phenotype in three-dimensional NMuMG epithelial-derived organoids indicating that p300 is required for EMT induction by TGFβ (Fig. 5C–E, S6B–D). Importantly, whereas expression of SnoN or SUMO-SnoN suppressed p300-induced EMT-like changes, SnoNKdR failed to promote EMT in p300-knockdown cell-derived organoids (Fig. 5B–E, S6B–D). In other experiments, the promotion of EMT upon expression of p300 required active endogenous/basal TGFβ signaling (Fig. S6E–H). Altogether, these data suggest that SnoN acts in a sumoylation-dependent manner to associate with p300 and thereby inhibits the ability of p300 to promote TGFβ-induced EMT-like behaviour in epithelial cell-derived organoids.

### Sumoylated SnoN regulates histone acetylation modulators to suppress TGFβ-induced EMT-related phenotypes in breast carcinoma organoids

**Fig. 6:**
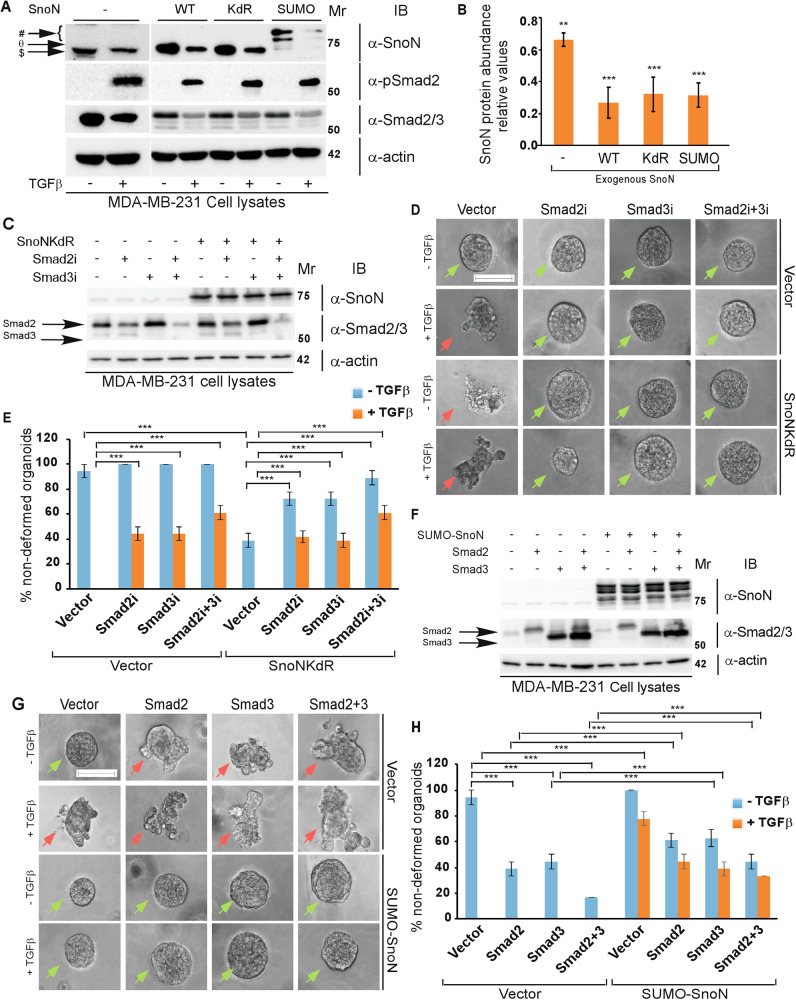
TGFβ-Smad2/3 pathway is a key target of SnoN suppression of EMT-associated responses in breast carcinoma organoids. **A**, **B** Impact of SnoN sumoylation status on reduction of SnoN protein level by TGFβ signaling. **A** SnoN, TGFβ-phosphorylated Smad2 on Serine residues 465 and 467 (pSmad2), Smad2/3, and actin (loading control) immunoblotting (IB) of lysates of MDA-MB-231 cells transfected with a stable vector control (−) or a plasmid stably expressing wild type SnoN (WT), SUMO loss of function SnoNKdR (KdR), or SUMO gain of function SUMO-SnoN (SUMO), incubated without (−) or with 100pM TGFβ (+) for 6 h. SnoN-immunoreactive protein species are indicated in the upper blot corresponding to endogenous SnoN ($ −120 s exposure), exogenous SnoN (WT or KdR) (Ө) and SUMO-SnoN (#) (5 s exposure), MDA-MB-231 cell extracts with (+) and without (−) exogenous TGFβ. **B** Bar graph depicts mean ± SEM of actin-normalized SnoN immunoreactive species intensity in lysates from one of the four TGFβ-treated MDA-MB-231 stables expressed relative to the untreated corresponding pair, obtained from three independent experiments including the one shown in 6 A. Statistical difference, Unpaired Student’s t-test: **P ≤ 0.01, ***P ≤ 0.00, each compared to its corresponding untreated stable cells. **C**–**E** *TGFβ-Smad pathway mediates SnoNKdR-induction of EMT in breast carcinoma organoids*. **C** SnoN, Smad2/3 and actin (loading control) immunoblotting (IB) of lysates of MDA-MB-231 cells transfected with a stable vector control (−) or plasmid stably expressing the SUMO loss of function SnoNKdR (+), with each transiently transfected with a control RNAi plasmid (−), or an RNAi plasmid expressing the Smad2-targetting shRNA Smad2i (+), the Smad3-targetting shRNA Smad3i (+), alone or together. **D** Representative differential interference contrast (DIC) light microscopy micrographs of untreated (−) or 100 pM TGFβ-treated (+) 8-day old organoids of MDA-MB-231 cells transfected and assessed as in **C**. **E** Bar graph depicts mean ± SEM proportion of non-deformed organoids expressed as a percentage of total colonies counted for each experimental condition from three biological replicates including the replicate with representative DIC images shown in **D**. **F**, **G** *SUMO-SnoN antagonizes Smad2/3-induced EMT in breast carcinoma organoids*. **F** SnoN, Smad2/3, and actin (loading control) immunoblotting (IB) of lysates of MDA-MB-231 cells transfected with a stable-vector control (−), or a plasmid stably expressing the SUMO gain of function SUMO-SnoN (+), with each transiently transfected with a vector control (−), or a plasmid encoding Smad2 (+) or Smad3 (+), alone or together. **G** Representative differential interference contrast (DIC) light microscopy micrographs of untreated or 100 pM TGFβ-treated 8-day old organoids of MDA-MB-231 cells transfected and assessed as in **F**. **H** Bar graph depicts mean ± SEM proportion of non-deformed organoids expressed as a percentage of total colonies counted for each experimental condition from three biological replicates including the replicate with the representative DIC images as shown in **G**. Green and red arrows indicate non-deformed and deformed organoids, respectively. Mr indicates Markers’ molecular size. Statistical difference, ANOVA: ***P ≤ 0.001. Scale bar indicates 50 μm.

**Fig. 7:**
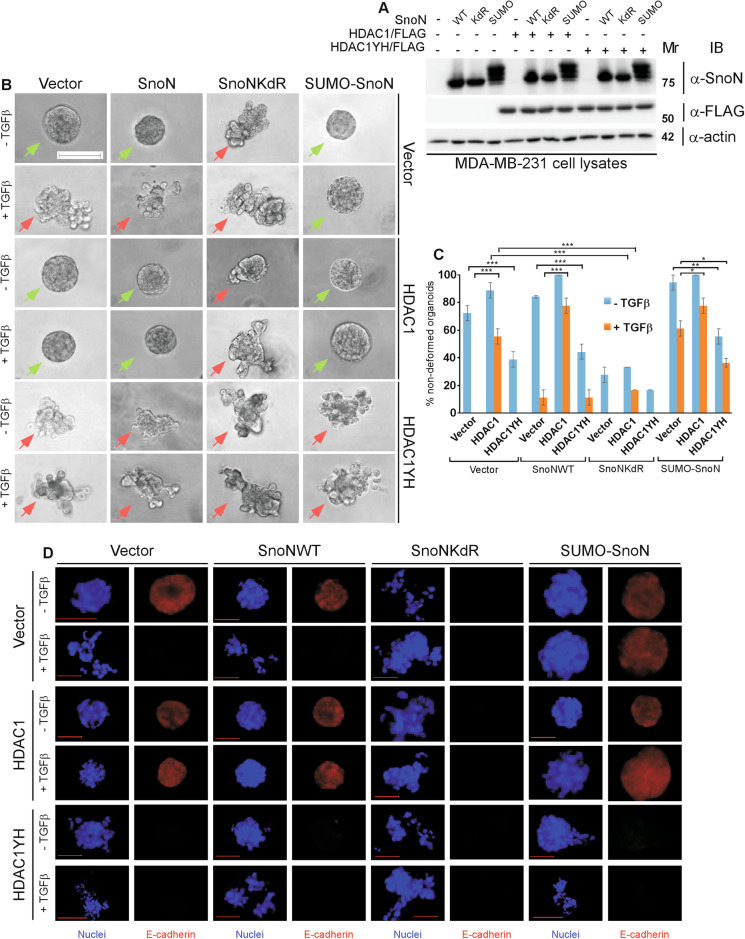
HDAC1 acts in a deacetylase-dependent manner to mediate sumoylated SnoN suppression of EMT in breast carcinoma organoids. **A** SnoN, HDAC1 and actin (loading control) immunoblotting (IB) of lysates of MDA-MB-231 cells transfected with a stable vector control (−), or a plasmid stably expressing the wild type SnoN (WT), the SUMO loss of function SnoNKdR (KdR), or the SUMO gain of function SUMO-SnoN (SUMO), with each transiently transfected with a vector control (−), or a plasmid encoding HDAC1/FLAG (+) or the deacetylase inactive HDAC1YH/FLAG ( + ). **B** Representative differential interference contrast (DIC) light microscopy micrographs of untreated (−) or 100 pM TGFβ-treated (+) 8-day old organoids of MDA-MB-231 cells transfected and assessed as in **A**. Green and red arrows indicate non-deformed and deformed organoids, respectively. **C** Bar graph depicts mean ± SEM proportion of non-deformed organoids expressed as a percentage of total colonies counted for each experimental condition from three biological replicates including the replicate with the representative DIC images as shown in **B**. **D** Representative fluorescence microscopy scans of E-cadherin-(immunocytochemistry-red) and nuclei-(Hoechst-blue) stained of fixed 8-day old organoids of MDA-MB-231 cells transfected and assessed as in **A**–**C**. Mr indicates Markers’ molecular size. Statistical difference, ANOVA: **P ≤ 0.01, ***P ≤ 0.001. Scale bar indicates 50 μm.

**Fig. 8:**
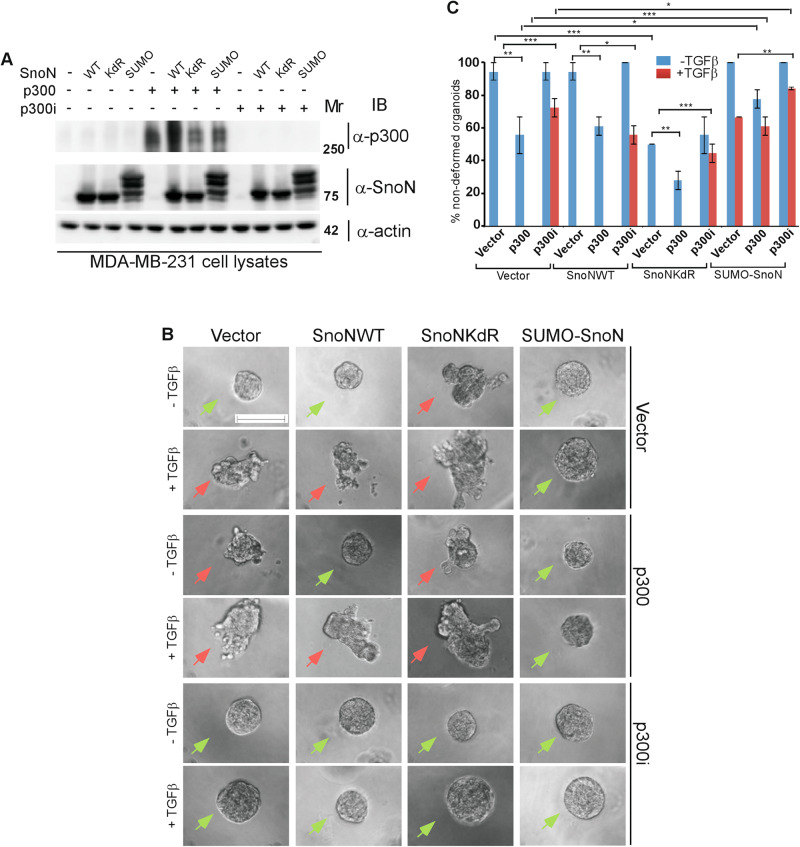
Sumoylated SnoN suppresses p300-mediated EMT in breast carcinoma organoids. **A** p300, SnoN and actin (loading control) immunoblotting (IB) of lysates of MDA-MB-231 cells transfected with a stable vector control (−), or a plasmid stably expressing the wild type SnoN (WT), the SUMO loss of function SnoNKdR (KdR), or the SUMO gain of function SUMO-SnoN (SUMO), with each transfected transiently with vector controls (−), or a plasmid encoding the protein p300/HA (+), or the p300-targetting shRNA p300i (+). **B** Representative differential interference contrast (DIC) light microscopy micrographs of untreated (−) or 100 pM TGFβ-treated (+) 8-day old organoids of MDA-MB-231 cells transfected and assessed as in A. Green and red arrows indicate non-deformed and deformed organoids, respectively. **C** Bar graph depicts mean ± SEM proportion of non-deformed organoids expressed as a percentage of total colonies counted for each experimental condition from three biological replicates including the replicate with representative DIC images shown in **B**. Mr indicates Markers’ molecular size. Statistical difference, ANOVA: *P ≤ 0.05, **P ≤ 0.01, ***P ≤ 0.001. Scale bar indicates 50 μm.

That SnoN associates in a sumoylation-dependent manner with HDAC1 and p300 raised the question of the functional consequences of this interaction in cancer cells (Figs. 3–5). EMT plays key roles in the invasion and metastasis of carcinomas including in breast cancer. SnoN acts in a sumoylation-dependent manner to suppress TGFβ-induced EMT-associated responses including invasive growth of human breast cancer cells [25, 26]. We asked whether sumoylated SnoN suppression of EMT in cancer cells is mediated by regulation of HDAC1 and/or p300 in the human triple negative breast cancer MDA-MB-231 cells. Activation of TGFβ signaling induces the ubiquitination and consequent degradation of SnoN in cells [38]. We first characterized if the ability of TGFβ signaling to reduce SnoN protein levels is sumoylation-dependent (Fig. 6A, B). Immunoblotting analyses in MDA-MB-231 cells suggested that SnoN’s sumoylation status has little or no effect on TGFβ signaling-mediated reduction in SnoN protein abundance (Fig. 6A, B). We next characterized the interplay between sumoylated SnoN and Smad2/3 in EMT in the MDA-MB-231 organoids. When cultured in the context of an extracellular matrix source, MDA-MB-231 cells organize into organoids showing a spherical phenotype with filled centre, reflecting the transformed nature of these cells [25, 26, 31, 33, 34]. TGFβ-induced EMT-associated morphogenetic changes manifested as deformation, budding and invasive growth (Figs. 6–8, S7, S8, S10), and loss of protein abundance of the epithelial marker E-cadherin, and cortical actin of MDA-MB-231 organoids (Fig. 7D, S8A, B, and [25, 34]) As expected, we found that expression of the SUMO loss of function SnoNKdR (Fig. 6C) promoted an EMT-associated response even in the absence of exogenous TGFβ in three-dimensional MDA-MB-231 cell-derived organoids (Fig. 6D, E). Knockdown of endogenous Smad2, Smad3, alone or together (Fig. 6C) suppressed TGFβ-induced EMT-like effects in MDA-MB-231 organoids (Fig. 6D, E). Importantly, knockdown of endogenous Smad2/3 reduced the ability of SnoNKdR to promote EMT in MDA-MB-231 organoids in the absence or presence of exogenous TGFβ (Fig. 6D, E). Conversely, expression of Smad2, Smad3, alone or together (Fig. 6F) promoted an EMT-like phenotype even in the absence of exogenous TGFβ in breast carcinoma organoids (Fig. 6G-H). Expression the SUMO gain of function SUMO-SnoN (Fig. 6F), as expected, suppressed the ability of exogenous TGFβ to induce EMT in breast carcinoma organoids (Fig. 6G-H) [25]. Importantly, SUMO-SnoN suppressed the ability of overexpressed Smad2 and Smad3 to promote EMT in breast carcinoma organoids (Fig. 6G-H). Together, these data suggest that SnoN acts in a Smad-dependent manner to regulate TGFβ-induced EMT responses in MDA-MB-231 breast carcinoma organoids (Fig. 6C-H).

Next, we investigated the role of HDAC1 on TGFβ-induced EMT in three-dimensional MDA-MB-231 breast carcinoma-derived organoids. Expression of HDAC1 (Fig. S7A), even modestly, decreased the EMT-associated invasive growth phenotype of MDA-MB-231 organoids in the absence or presence of exogenous TGFβ (Fig. S7B, C). Conversely, knockdown of endogenous HDAC1 (Fig. S7A) promoted an EMT-like phenotype in breast carcinoma organoids even in the absence of exogenous TGFβ (Fig. S7B, C). Expression of the sumoylation-defective SnoNKdR (Fig. S7A) reduced the ability of HDAC1 to suppress TGFβ-induced EMT-associated invasive growth of the breast carcinoma-derived organoids (Fig. S7B, C). On the other hand, endogenous HDAC1 knockdown (Fig. S7A) reduced the ability of SUMO-SnoN to suppress TGFβ-induced EMT-like phenotype of the breast carcinoma organoids (Fig. S7B, C). Together, these data suggest that sumoylated SnoN acts in an HDAC1-dependent manner to suppress TGFβ-Smad-induced EMT in breast carcinoma organoids.

We next determined the role of the histone deacetylase activity of HDAC1 in suppression of EMT-associated effects in breast carcinoma organoid. Expression of the enzymatically inactive HDAC1YH (Fig. 7A) phenocopied HDAC1 knockdown in promoting EMT, as indicated by the invasive growth and loss of the protein abundance of the epithelial marker E-cadherin, and loss of the cortical actin organization phenotypes in three-dimensional MDA-MB-231 cell-derived organoids, even in the absence of exogenous TGFβ (Fig. 7B–D, S8A, B). Importantly, HDAC1YH decreased SUMO-SnoN suppression of EMT in breast carcinoma organoids (Fig. 7A–D, S8A, B). These data suggest that the deacetylase activity of HDAC1 is required for SnoN to suppress TGFβ-induced EMT behaviour in breast carcinoma organoids (Fig. 7A-D, S8A-B). In other analyses, promotion of EMT by RNAi-induced knockdown of HDAC1 and expression of the dominant negative deacetylase HDAC1YH required endogenous/basal TGFβ-Smad signaling in the MDA-MB-231 organoids (Fig. S8C–F). Increased cell migration represents a feature of EMT. We therefore characterized the role of sumoylation in the effect of the SnoN-HDAC1 complex on MDA-MB-231 cell monolayers migration, in the absence and presence of exogenous TGFβ using the scratch assay (Fig. S9A, B). As expected, exogenous TGFβ promoted MDA-MB-231 cell migration (Fig. S9A, B). Expression of HDAC1 suppressed, whereas deacetylase-inactive HDAC1YH or knockdown of endogenous HDAC1 promoted TGFβ-induced migration of MDA-MB-231 cells (Fig. S9A, B). We also found that expression of SnoNKdR promoted, whereas SUMO-SnoN suppressed TGFβ-induced MDA-MB-231 cell migration (Fig. S9A, B). Importantly, SnoNKdR reversed HDAC1 suppression of migration, and conversely SUMO-SnoN reduced the ability of HDAC1YH or knockdown of endogenous HDAC1 to promote migration of these cells (Fig. S9A, B). These data further support the conclusion that the sumoylated SnoN-HDAC1 axis plays a key role in suppression of TGFβ-induced EMT-associated effects (Fig. S9A, B). Collectively, these findings suggest that sumoylation enhances the SnoN-HDAC1 association, and in turn suppresses TGFβ-induced EMT and associated responses in breast cancer cells.

We also determined the functional consequence of sumoylated SnoN-p300 interaction in in the context of EMT in breast carcinoma organoids. Expression of p300 promoted, whereas knockdown of p300 suppressed, EMT morphogenetic changes in MDA-MB-231 organoids in the absence or presence of exogenous TGFβ (Fig. 8A-C). Importantly, we found that overexpression of SUMO-SnoN suppressed the ability of p300 expression to induce EMT-like morphogenetic changes in the breast carcinoma organoids (Fig. 8A–C). Conversely, knockdown of p300 abrogated the ability of SnoNKdR to promote EMT in MDA-MB-231 carcinoma organoids (Fig. 8A–C). In other studies, the ability of expressed p300 to promote EMT-like effects in the MDA-MB-231 organoids required endogenous/basal TGFβ-Smad signaling (Fig. S10A–C). We also characterized the functional consequences of the SnoN-p300 complex in regulating EMT using MDA-MB-231 cell migration assays (Fig. S11A, B). We found that p300 expression promoted, whereas p300 knockdown suppressed the ability of TGFβ to enhance migration of MDA-MB-231 cells (Fig. S11A, B). Interestingly, expression of SnoNKdR rescued the reduction in cell migration upon p300-knockdown (Fig. S11A, B). Conversely, SUMO-SnoN abrogated the ability of expressed p300 to promote MDA-MB-231 cell migration (Fig. S11A, B). Together, these data suggest that p300 promotes TGFβ-induced EMT-associated morphogenetic phenotype and responses in breast cancer cells. In addition, SnoN sumoylation promotes SnoN-p300 association to suppress EMT in breast carcinomas.

Collectively, our study reveals that sumoylation promotes the ability of SnoN to associate with the histone acetylation modulators HDAC1 and p300 with functional implications for SnoN suppression of TGFβ-Smad-induced EMT in mammary epithelial and carcinoma organoids. These findings add insights to the molecular mechanisms by which the SUMO pathway regulates biological processes in normal and disease states.

## Discussion

In this study, we have uncovered a novel link between the SUMO pathway and modulators of histone acetylation with important implications for regulation of EMT in epithelial and carcinoma organoids. We find that sumoylation of the transcription coregulator SnoN promotes its association with the histone deacetylase HDAC1 and the acetyl transferase p300. Endogenous HDAC1 suppresses, whereas endogenous p300 promotes EMT in epithelial and carcinoma organoids. Importantly, we find that SnoN acts in a sumoylation-dependent fashion to stimulate and inhibit, respectively, the effects of HDAC1 and p300 on EMT-associated morphological and functional changes in breast epithelial and carcinoma cells. Our findings should advance our understanding of the biology of the TGFβ and sumoylation pathways.

The discovery that sumoylation promotes the ability of SnoN to associate with HDAC1 and p300 with opposing effects on their ability to regulate EMT provides mechanistic insights into how this modification enables SnoN to regulate EMT. These results help explain the critical role that sumoylation plays in SnoN suppression of Smad-mediated EMT. It will be interesting in future studies to characterize whether sumoylated SnoN regulates the proportion of Smad-HDAC1- and Smad-p300-containg protein complexes in cells.

The finding that HDAC1 acts in a deacetylase-dependent manner to suppress TGFβ-Smad signaling-mediated EMT in the context of epithelial organoids contributes to better understanding of the biology of HDAC1. HDAC1 acts with distinct transcription factors to regulate the abundance of mRNA encoding E-cadherin to promote or suppress EMT [41–43]. Interestingly, incubation of human adrenal cortical carcinoma cells with a class I HDAC inhibitor promotes EMT in these cells, supporting the idea that HDAC1 may act as a suppressor of EMT [44]. That HDAC1 suppresses EMT in a deacetylase-dependent manner, uncovered in the current study, is further supported by our finding that the histone acetylase p300 promotes EMT in epithelial and carcinoma organoids, as well as increased TGFβ-induced breast cancer cell migration, a key functional consequence of EMT. The protein p300 is thought to promote EMT in different tissue systems [16, 45–47]. p300 has been associated with cancer progression and poor prognosis in a number of tumor types [45–47], including breast cancer [48, 49]. Recently an inhibitor of p300-mediated Snail acetylation was reported to inhibit the growth and metastasis of p53-wild-type tumors [50]. It will be interesting to explore the extent of the antagonistic relationship between HDAC1 and p300 in controlling EMT in normal development and cancer pathogenesis.

The idea that sumoylated SnoN enables HDAC1 to act in a deacetylase-dependent manner to regulate TGFβ-Smad-induced EMT has implications for the regulation of EMT. That the activity of HDAC1 is critical for sumoylated SnoN to suppress TGFβ-induced EMT in non-transformed and transformed mammary epithelial organoids adds mechanistic insights into how posttranslational modification of SnoN can impact the ability of epigenetic regulators to modulate cellular processes. The SUMO loss of function SnoN, SnoNKdR has been shown to phenocopy endogenous SnoN knockdown in promoting EMT [14, 24, 32, 51]. Thus, that expression of SnoNKdR reduces the ability of HDAC1 to suppress TGFβ-induced EMT suggests that HDAC1 suppression of EMT is regulated by the sumoylation status of endogenous SnoN. The lower HDAC1 binding capacity of non-sumoylated SnoN as compared to the sumoylated species may reduce HDAC1-containing Smad transcriptional regulatory complex with positive impact on EMT. TGFβ-Smad signaling recruits distinct E3 ubiquitin-ligases including Smurf2, Arkadia and APC-Cdh1 to SnoN leading to its ubiquitination and consequent proteasomal degradation in different cell types [38, 52, 53]. It would be important to address if such mechanisms are involved in TGFβ regulation of SnoN stability in the cellular models described here in future studies. Such future studies can be designed to characterize cell and context-based differences in the regulation of the protein abundance of SnoN by TGFβ signaling, and specific molecular mechanisms involved and biological significance. Our finding that TGFβ-reduction of SnoN protein level in breast cancer cells may not be affected by the sumoylation status of SnoN raises the key question whether TGFβ signaling may act via other mechanisms to differentially regulate the abundance of sumoylated forms of SnoN in cells. Consistent with this idea, TGFβ signaling has been shown to reduce the proportion of sumoylated species of SnoN to a greater extent than the unmodified form, by promoting the ubiquitin-mediated degradation of the SUMO E3 ligase PIAS1 in cells undergoing EMT [14, 26]. Overall, the potential differential reduction in sumoylated SnoN by TGFβ signaling may disinhibit the effect HDAC1 on EMT in cells. Because SnoN recruits and forms a multiprotein complex with PIAS1 and TIF1γ, the SUMO E3 ligase-complex may a play role in HDAC1’s ability to suppress EMT.

The discovery that SnoN acts in a sumoylation-dependent manner to bind and differentially regulate HDAC1 and p300 opposing roles in EMT suggest that this modification provides a double-edged sword in suppressing this fundamental cellular process. It would thus be interesting to determine in future studies whether p300 and HDAC1 influence each other’s association with SnoN. In addition, because HDAC1 and p300 form higher-order protein complexes including the epigenetic regulators Sin3A, NCoR, and the histone demethylases SETDB1, it will be interesting to investigate the possible physical and functional association of SnoN with these epigenetic factors in non-transformed and transformed cells derived from epithelial tissues in the context of EMT and other cellular responses.

The critical role of TGFβ regulation of EMT plays in development and cancer highlights the importance of understanding the molecular mechanisms by which EMT is regulated. Our findings add significant insights into how the SUMO pathway contributes to the regulation of EMT in epithelial cells.

## Supplementary information

Supplementary Figures

Supplementary Figure Legends

Reproducibility checklist

## Data Availability

All data are provided within the main text, figures, or supplementary materials. In addition, these analyses and original data are available from the corresponding author upon reasonable request.
