# Supplementary material for: Sumoylated SnoN interacts with HDAC1 and p300/CBP to regulate EMT-associated phenotypes in mammary organoids
Source: Cell Death Dis. 2023 Jul 7;14(7):405. doi: 10.1038/s41419-023-05921-x (PMC10326038; doi:10.1038/s41419-023-05921-x)

Figure S1

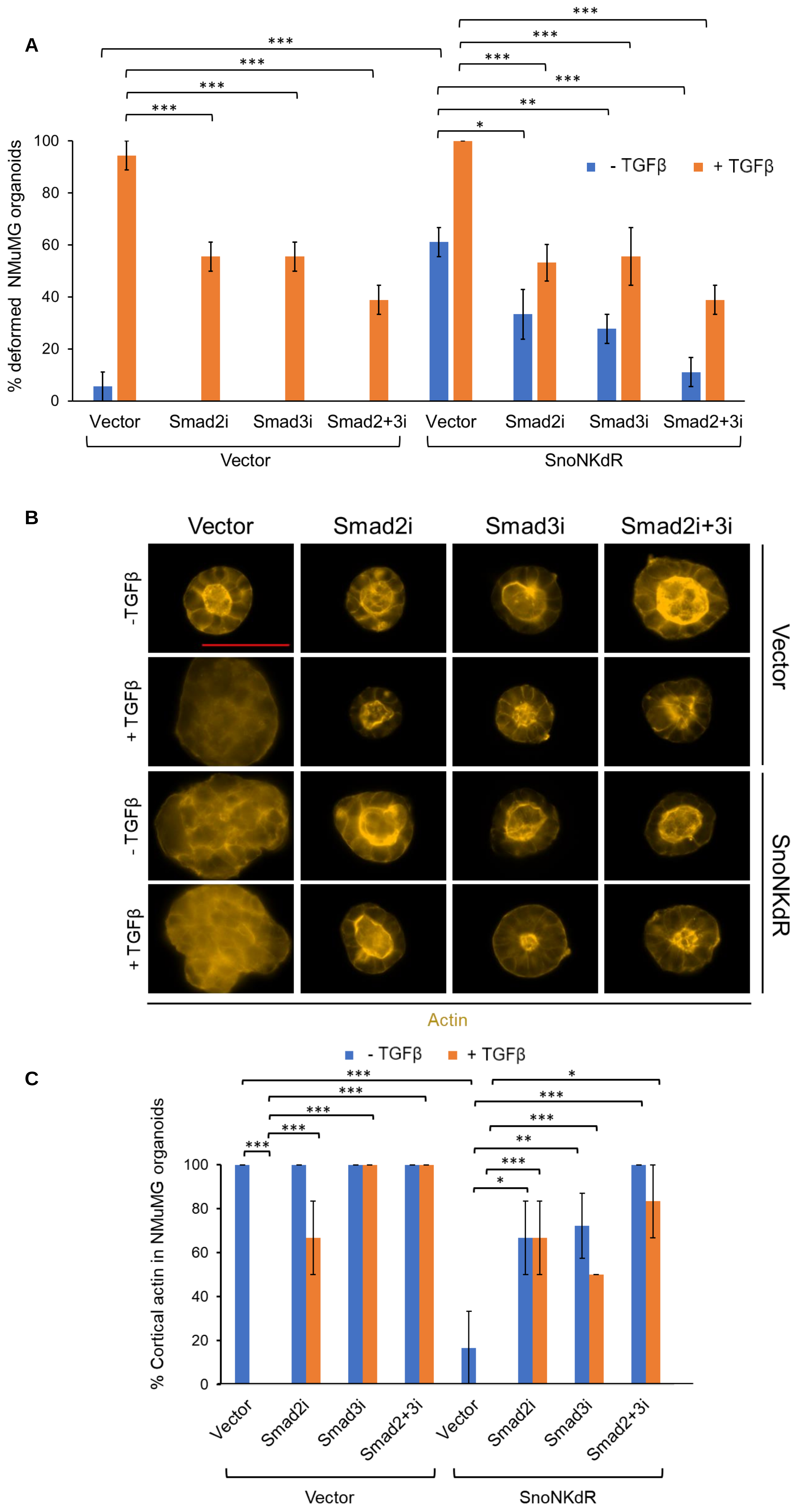
A
% deformed NMuMG organoids
- TGFβ
+ TGFβ
Vector
Smad2i
Smad3i
Smad2+3i
Vector
SnoNKdR
B
Vector
Smad2i
Smad3i
Smad2i+3i
-TGFβ
+ TGFβ
- TGFβ
+ TGFβ
Vector
SnoNKdR
Actin
C
% Cortical actin in NMuMG organoids
- TGFβ
+ TGFβ
Vector
Smad2i
Smad3i
Smad2+3i
Vector
SnoNKdR

Figure S2

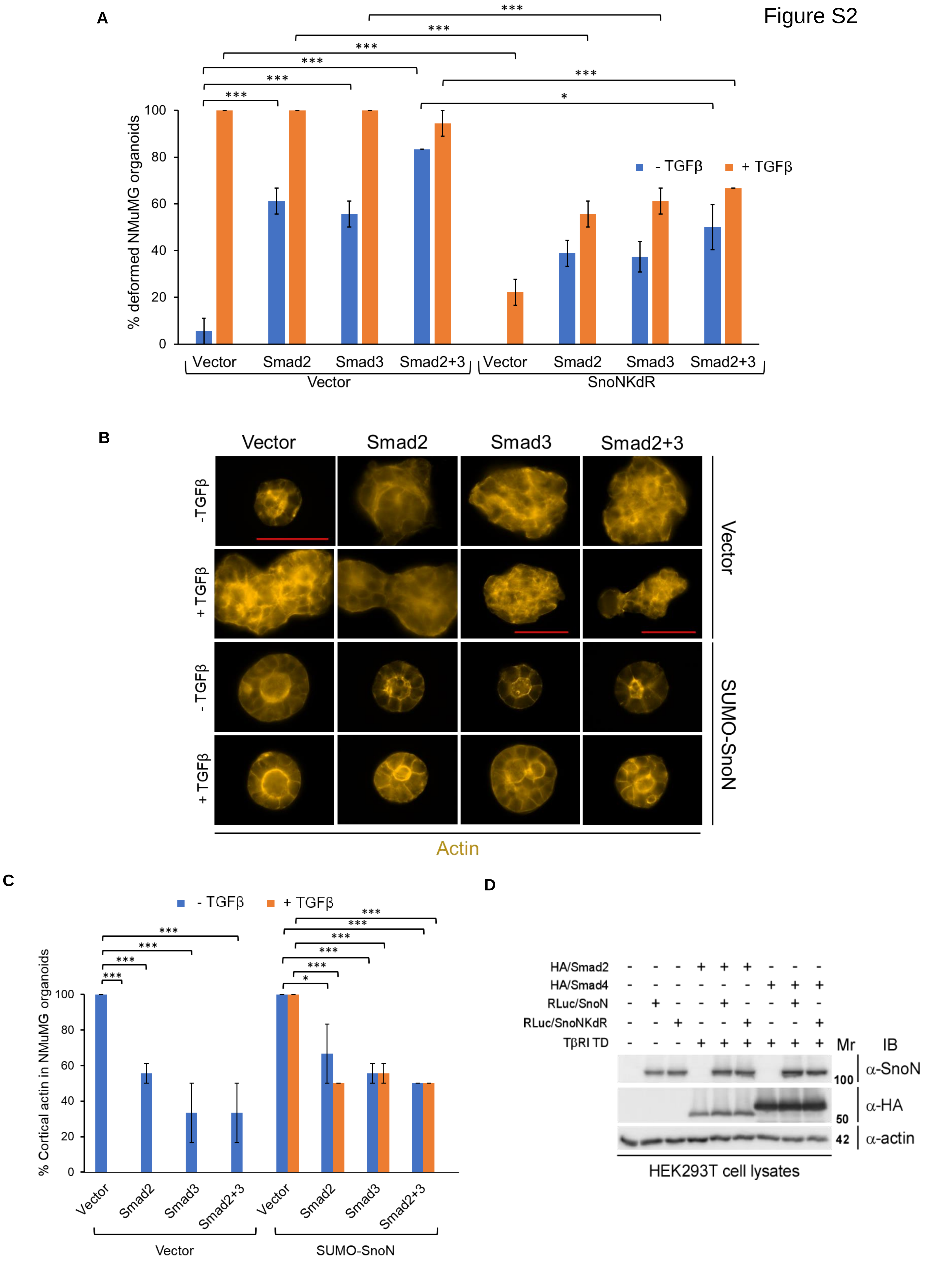

A
% deformed NMuMG organoids
- TGFβ
+ TGFβ
Vector
Smad2
Smad3
Smad2+3
Vector
SnoNKdR
B
Vector
Smad2
Smad3
Smad2+3
-TGFβ
+ TGFβ
- TGFβ
+ TGFβ
Vector
SUMO-SnoN
Actin
C
- TGFβ
+ TGFβ
% Cortical actin in NMuMG organoids
Vector
Smad2
Smad3
Smad2+3
Vector
SUMO-SnoN
D
HA/Smad2
HA/Smad4
RLuc/SnoN
RLuc/SnoNKdR
TβRI TD
Mr
IB
α-SnoN
α-HA
α-actin
100
50
42
HEK293T cell lysates

Figure S3

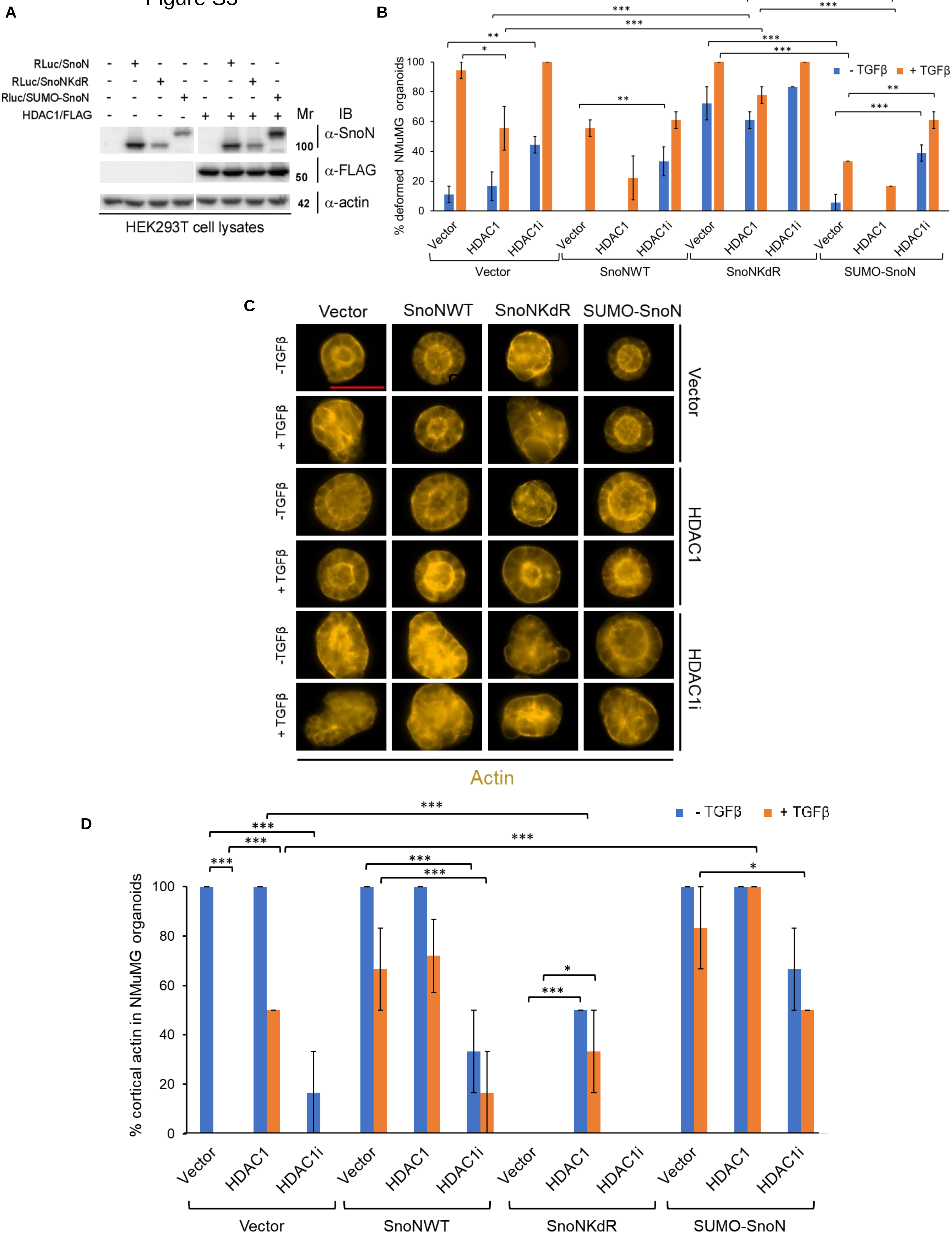

A
RLuc/SnoN
RLuc/SnoNKdR
Rluc/SUMO-SnoN
HDAC1/FLAG
Mr
IB
α-SnoN
α-FLAG
α-actin
100
50
42
HEK293T cell lysates
B
% deformed NMuMG organoids
- TGFβ
+ TGFβ
Vector
HDAC1
HDAC1i
Vector
SnoNWT
SnoNKdR
SUMO-SnoN
C
Vector
SnoNWT
SnoNKdR
SUMO-SnoN
-TGFβ
+ TGFβ
Vector
HDAC1
HDAC1i
Actin
D
% cortical actin in NMuMG organoids
- TGFβ
+ TGFβ
Vector
HDAC1
HDAC1i
Vector
SnoNWT
SnoNKdR
SUMO-SnoN

Figure S4

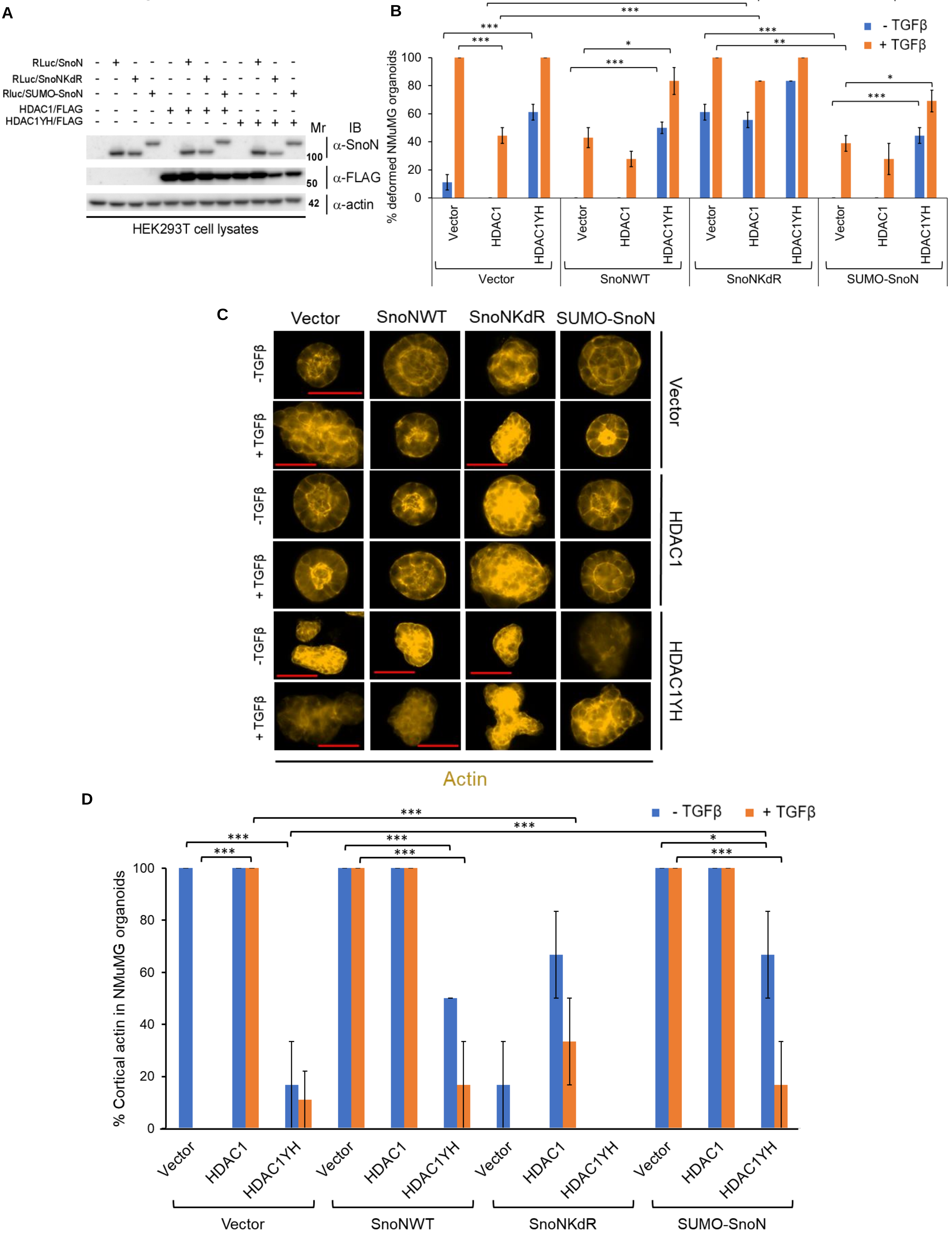

A
RLuc/SnoN
RLuc/SnoNKdR
Rluc/SUMO-SnoN
HDAC1/FLAG
HDAC1YH/FLAG
Mr
IB
α-SnoN
α-FLAG
α-actin
100
50
42
HEK293T cell lysates
B
- TGFβ
+ TGFβ
% deformed NMuMG organoids
Vector
HDAC1
HDAC1YH
Vector
SnoNWT
SnoNKdR
SUMO-SnoN
C
Vector
SnoNWT
SnoNKdR
SUMO-SnoN
-TGFβ
+ TGFβ
Vector
HDAC1
HDAC1YH
Actin
D
- TGFβ
+ TGFβ
% Cortical actin in NMuMG organoids
Vector
HDAC1
HDAC1YH
Vector
SnoNWT
SnoNKdR
SUMO-SnoN

Figure S5

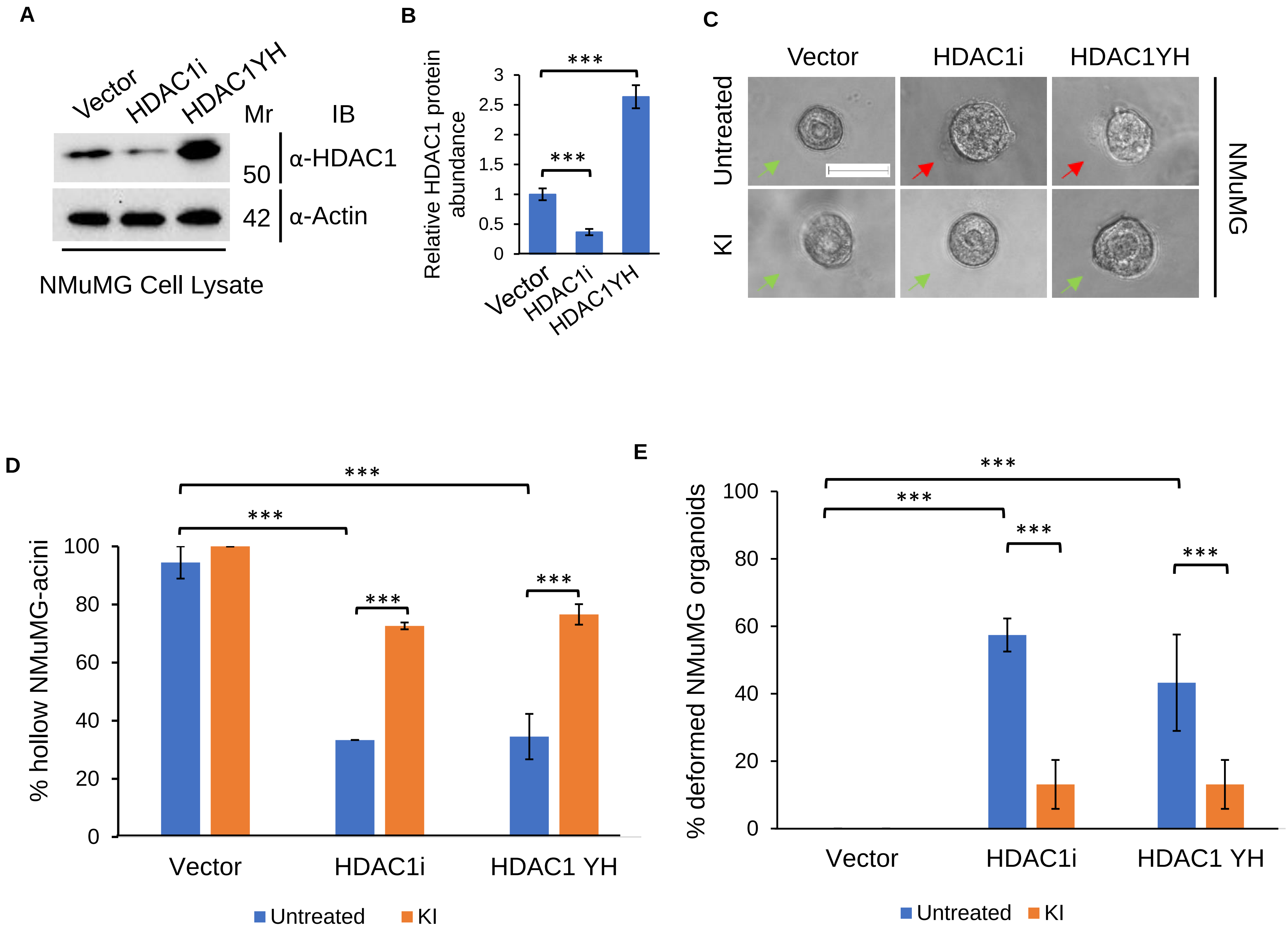
A
Vector
HDAC1i
HDAC1YH
Mr
IB
50
α-HDAC1
42
α-Actin
NMuMG Cell Lysate
B
Relative HDAC1 protein abundance
3
2.5
2
1.5
1
0.5
0
***
***
Vector
HDAC1i
HDAC1YH
C
Vector
HDAC1i
HDAC1YH
Untreated
KI
NMuMG
D
% hollow NMuMG-acini
100
80
60
40
20
0
***
***
***
***
Vector
HDAC1i
HDAC1 YH
Untreated
KI
E
% deformed NMuMG organoids
100
80
60
40
20
0
***
***
***
***
Vector
HDAC1i
HDAC1 YH
Untreated
KI

Figure S6

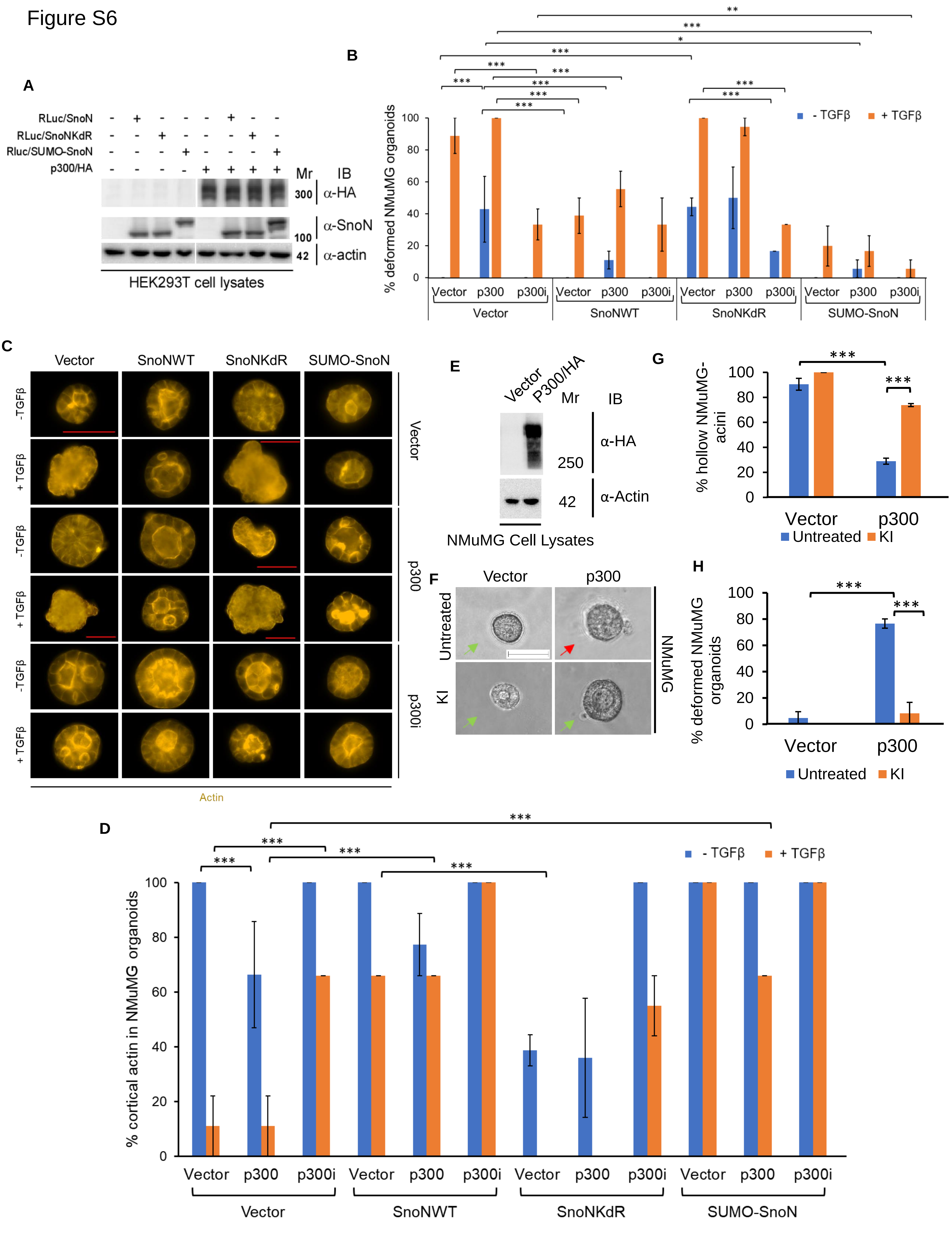
A
RLuc/SnoN
RLuc/SnoNKdR
Rluc/SUMO-SnoN
p300/HA
Mr
IB
300
α-HA
α-SnoN
100
42
α-actin
HEK293T cell lysates
B
% deformed NMuMG organoids
- TGFβ
+ TGFβ
Vector
p300
p300i
Vector
SnoNWT
SnoNKdR
SUMO-SnoN
C
Vector
SnoNWT
SnoNKdR
SUMO-SnoN
-TGFβ
+ TGFβ
Vector
p300
p300i
Actin
E
Vector
P300/HA
Mr
IB
α-HA
250
42
α-Actin
NMuMG Cell Lysates
F
Vector
p300
Untreated
KI
NMuMG
G
% hollow NMuMG-acini
Vector
p300
Untreated
KI
H
% deformed NMuMG organoids
Vector
p300
Untreated
KI
D
% cortical actin in NMuMG organoids
- TGFβ
+ TGFβ
Vector
p300
p300i
Vector
SnoNWT
SnoNKdR
SUMO-SnoN

Figure S7

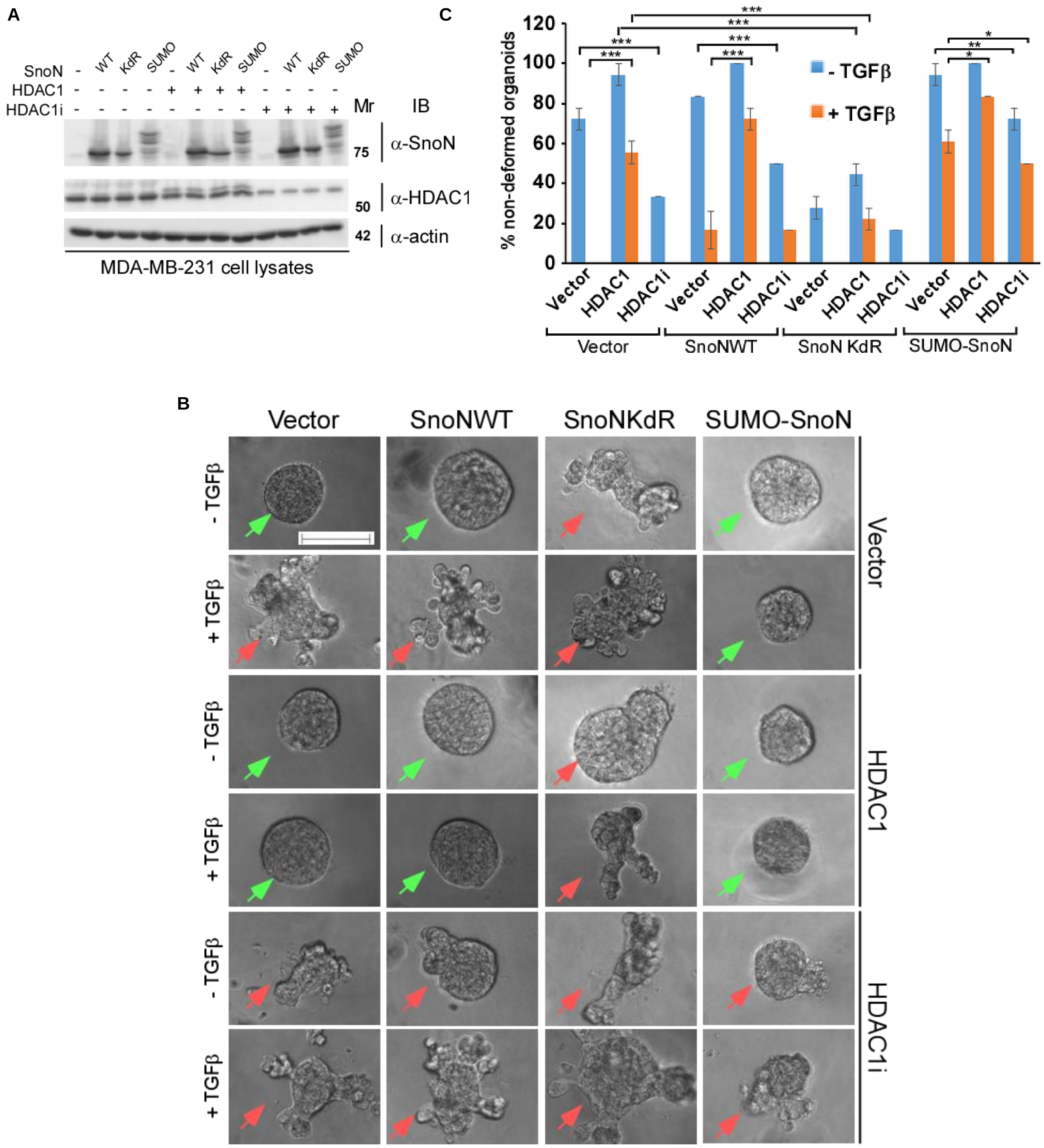
A
SnoN - WT KdR SUMO - WT KdR SUMO - WT KdR SUMO
HDAC1 - - - - + + + + - - - -
HDAC1i - - - - - - - - + + + +
Mr
IB
75
α-SnoN
50
α-HDAC1
42
α-actin
MDA-MB-231 cell lysates
B
Vector
SnoNWT
SnoNKdR
SUMO-SnoN
- TGFβ
+ TGFβ
Vector
HDAC1
HDAC1i
C
% non-deformed organoids
0
20
40
60
80
100
120
- TGFβ
+ TGFβ
***
**
*
Vector
HDAC1
HDAC1i
SnoNWT
SnoN KdR
SUMO-SnoN

Figure S8

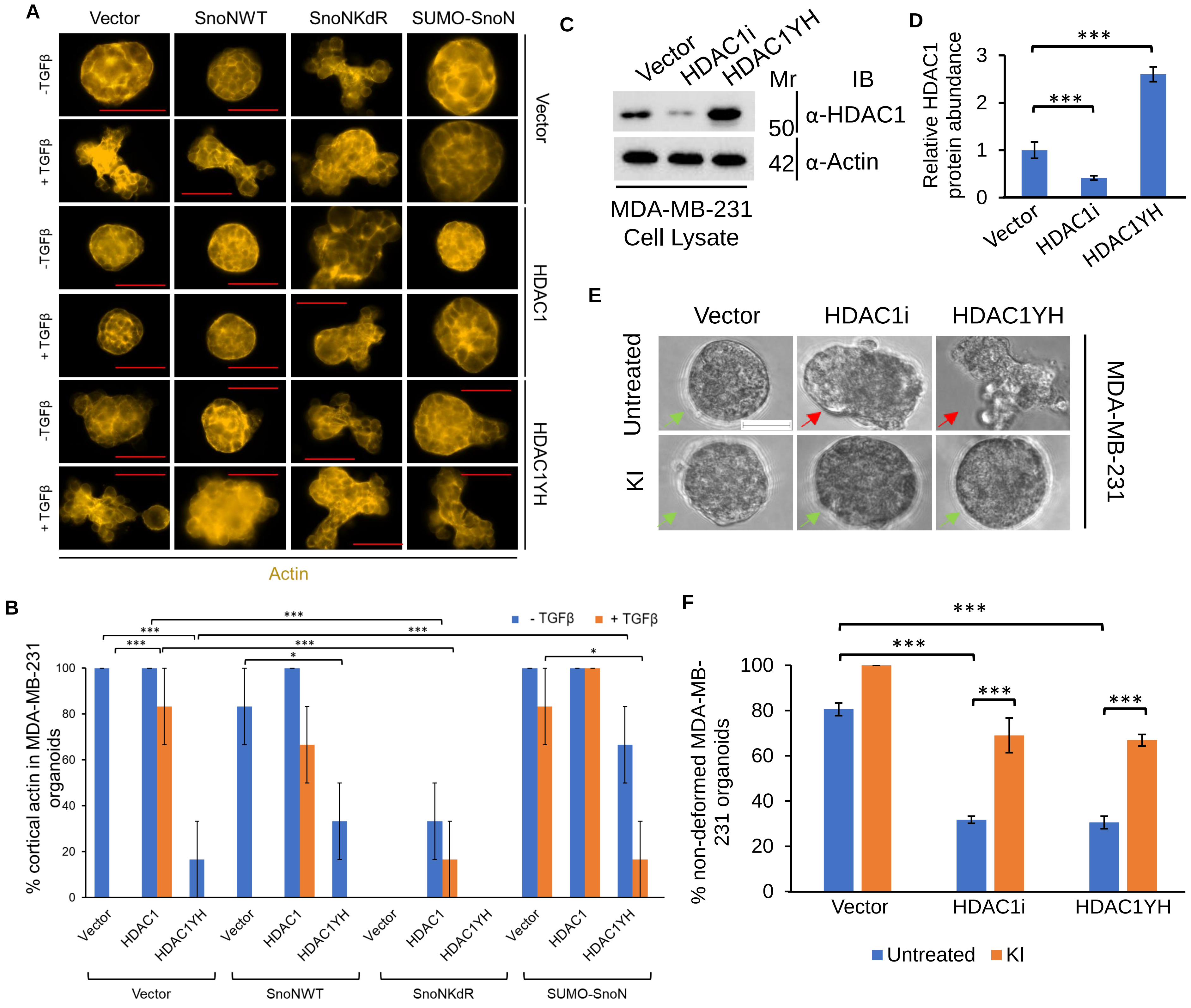

A
Vector
SnoNWT
SnoNKdR
SUMO-SnoN
-TGFβ
+ TGFβ
Vector
HDAC1
HDAC1YH
Actin
B
% cortical actin in MDA-MB-231 organoids
- TGFβ
+ TGFβ
***
*
SnoNWT
SnoNKdR
SUMO-SnoN
C
Vector
HDAC1i
HDAC1YH
Mr
IB
50
α-HDAC1
42
α-Actin
MDA-MB-231 Cell Lysate
D
Relative HDAC1 protein abundance
E
Untreated
KI
MDA-MB-231
F
% non-deformed MDA-MB-231 organoids
Untreated
KI

Figure S9

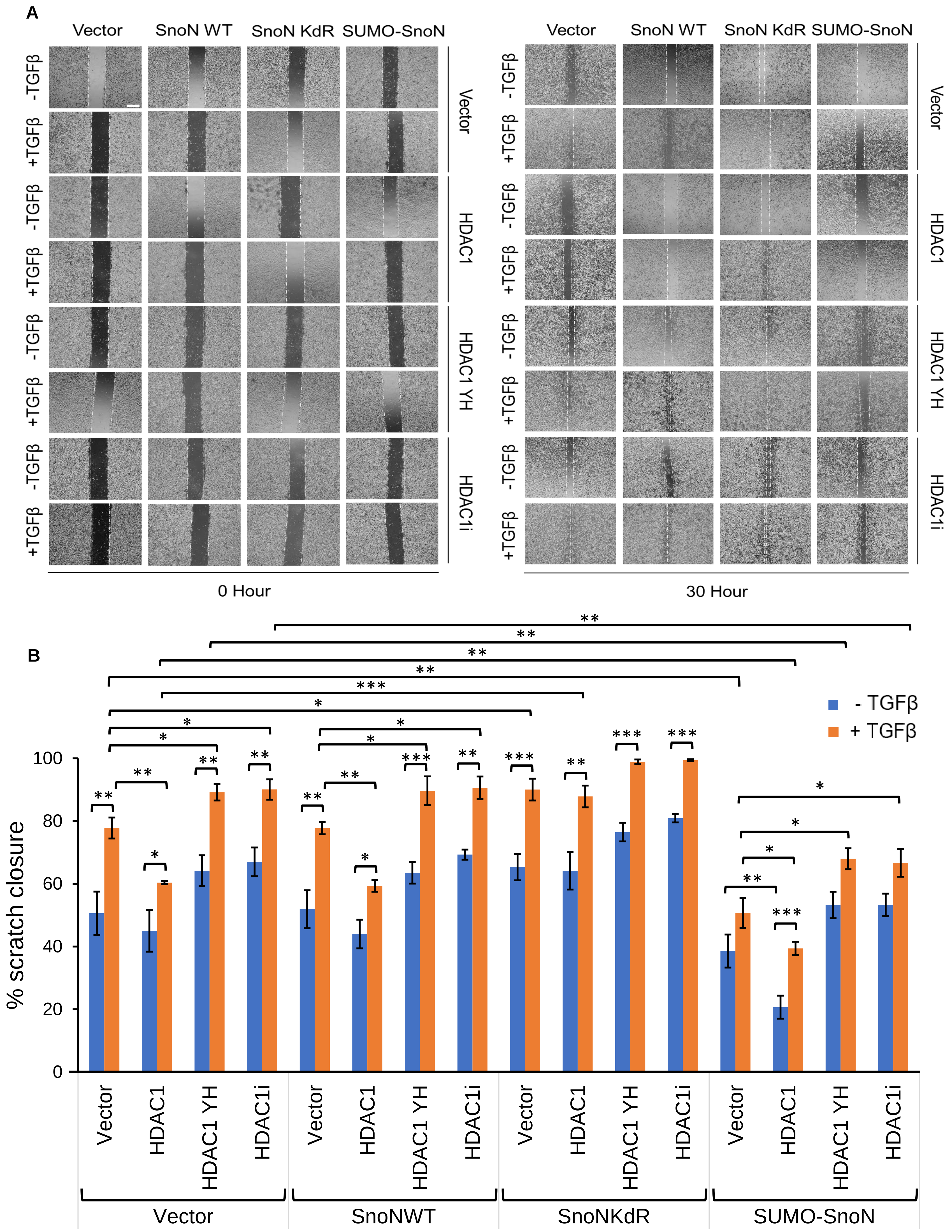
A
Vector
SnoN WT
SnoN KdR
SUMO-SnoN
-TGFβ
+TGFβ
Vector
HDAC1
HDAC1 YH
HDAC1i
0 Hour
30 Hour
B
- TGFβ
+ TGFβ
% scratch closure
100
80
60
40
20
0
Vector
HDAC1
HDAC1 YH
HDAC1i
Vector
SnoNWT
SnoNKdR
SUMO-SnoN

Figure S10

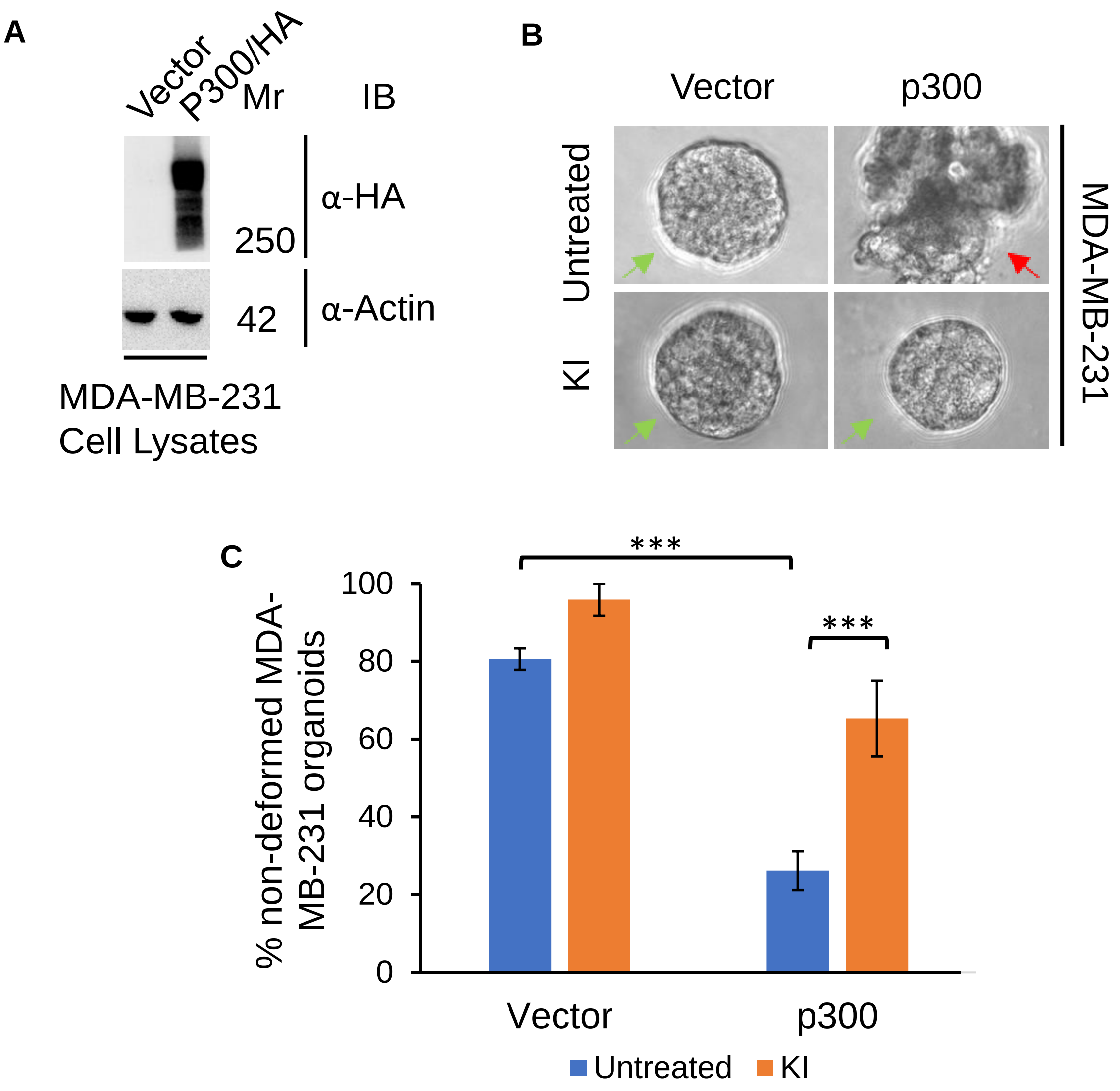

A
Vector
P300/HA
Mr
IB
250
α-HA
42
α-Actin
MDA-MB-231
Cell Lysates
B
Vector
p300
Untreated
KI
MDA-MB-231
C
% non-deformed MDA-MB-231 organoids
100
80
60
40
20
0
***
***
Vector
p300
Untreated
KI

Figure S11

A

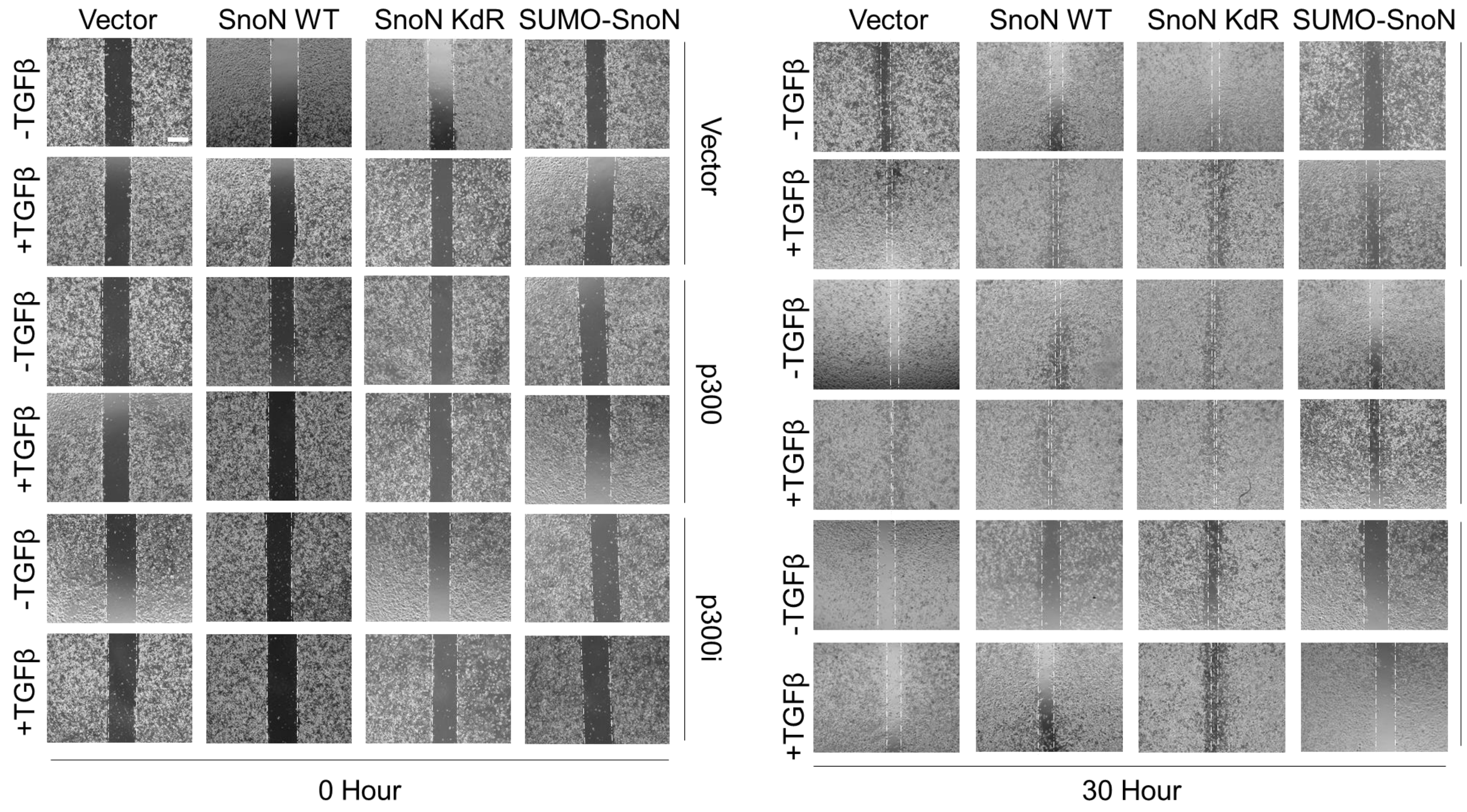

B

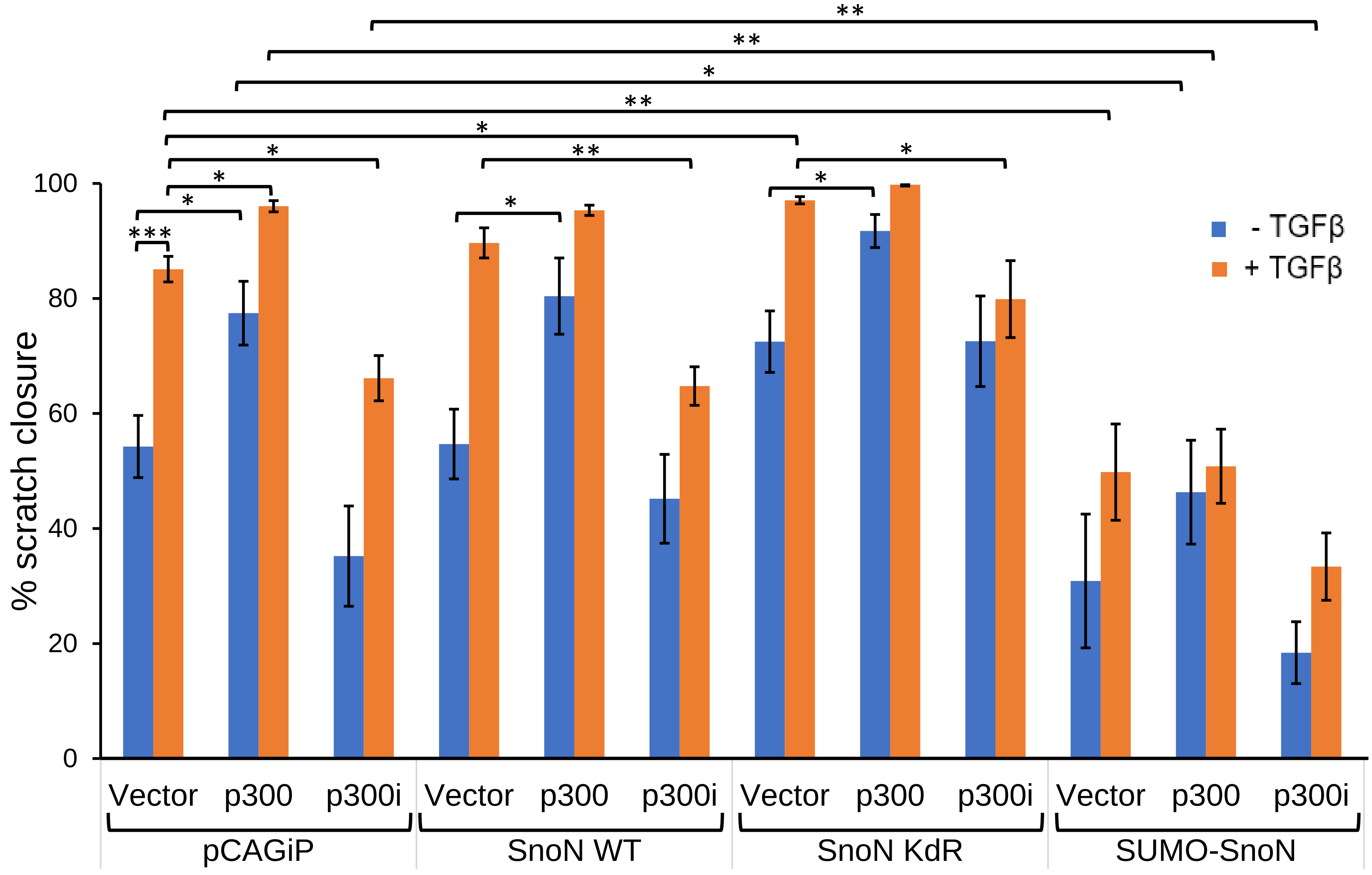

Supplement: Supplementary file 1 — Supplementary Figures [file 41419_2023_5921_MOESM1_ESM.pdf]
