# Supplementary material for: Sumoylated SnoN interacts with HDAC1 and p300/CBP to regulate EMT-associated phenotypes in mammary organoids
Source: Cell Death Dis. 2023 Jul 7;14(7):405. doi: 10.1038/s41419-023-05921-x (PMC10326038; doi:10.1038/s41419-023-05921-x)
Supplement: Supplementary file 2 — Supplementary Figure Legends [file 41419_2023_5921_MOESM2_ESM.docx]

**Figure S1: Related to Figure 1.**

A) Bar graph depicts mean ± SEM proportion of deformed NMuMG organoids expressed as a percentage of total colonies scored for each experimental condition from three biological replicates including the one with representative differential interference contrast (DIC) images shown in 1B. B) Representative fluorescence microscopy scans of Actin-(TRITC-phalloidin-orange) stained fixed 8-day old organoids of NMuMG cells transfected and assessed as in 1A-C. Scale bar indicates 50 μm. C) Bar graph depicts mean ± SEM proportion of cortical actin organization expressed as a percentage of total colonies scored for each experimental condition from three biological replicates including the one with representative IF images shown in B. Statistical difference, ANOVA: *P≤0.05, **P≤0.01, ***P≤0.001.

**Figure S2: Related to Figure 2.**

A) Bar graph depicts mean ± SEM proportion of deformed NMuMG organoids expressed as a percentage of total colonies scored for each experimental condition from three biological replicates including the one with representative differential interference contrast (DIC) images shown in 2B. B) Representative fluorescence microscopy scans of Actin-(TRITC-phalloidin-orange) stained fixed 8-day old organoids of NMuMG cells transfected and assessed as in 2A-C. Scale bars indicate 50 μm. C) Bar graph depicts mean ± SEM proportion of cortical actin organization expressed as a percentage of total colonies scored for each experimental condition from three biological replicates including the one with representative IF images shown in B. Statistical difference, ANOVA: *P≤0.05, **P≤0.01, ***P≤0.001. D) SnoN, Smad2/4 (HA) and actin (loading control) immunoblotting (IB) of 20% of lysates of cells transfected with a plasmid encoding only the Renilla luciferase protein (RLuc) (-), or in fusion with wild type SnoN (RLuc/SnoN) (+), or the SUMO loss of function SnoNKdR (RLuc/SnoNKdR) (+), together with a vector control (-), or with a plasmid encoding HA/Smad2 (+) or HA/Smad4 (+), and a plasmid encoding a constitutively active TGFβ type I receptor (TβRI TD) (+). The immunoblotting analyses are input data relating to Figure 2E. Mr indicates Markers’ molecular size.

**Figure S3: Related to Figure 3.**

A) SnoN, HDAC1 (FLAG) and actin (loading control) immunoblotting (IB) of 20% of lysates of cells transfected with a plasmid encoding only the Renilla luciferase protein (RLuc) (-), or in fusion with wild type SnoN (RLuc/SnoN) (+), the SUMO loss of function SnoNKdR (RLuc/SnoNKdR) (+), or the SUMO gain of function SUMO-SnoN (RLuc/SUMO-SnoN) (+), together with a vector control (-), or a plasmid encoding HDAC1/FLAG (+). The immunoblotting analyses are input data relating to Figure 3A. Mr indicates Markers’ molecular size. B) Bar graph depicts mean ± SEM proportion of deformed NMuMG organoids expressed as a percentage of total colonies scored for each experimental condition from three biological replicates including the one with representative differential interference contrast (DIC) images shown in 3C. C) Representative fluorescence microscopy scans of Actin-(TRITC-phalloidin-orange) stained fixed 8-day old organoids of NMuMG cells transfected and assessed as in 3B-D. Scale bar indicates 50 μm. D) Bar graph depicts mean ± SEM proportion of cortical actin organization expressed as a percentage of total colonies scored for each experimental condition from three biological replicates including the one with representative IF images shown in C. Statistical difference, ANOVA: *P≤0.05, **P≤0.01, ***P≤0.001.

**Figure S4: Related to Figure 4.**

A) SnoN, HDAC1/HDAC1YH (FLAG) and actin (loading control) immunoblotting (IB) of 20% of lysates of cells transfected with a plasmid encoding only the Renilla luciferase protein (RLuc) (-), or in fusion with wild type SnoN (RLuc/SnoN) (+), the SUMO loss of function SnoNKdR (RLuc/SnoNKdR) (+), or the SUMO gain of function SUMO-SnoN (RLuc/SUMO-SnoN) (+), together with a vector control (-), or a plasmid encoding HDAC1/FLAG (+) or the deacetylase-inactive HDAC1YH/FLAG. The immunoblotting analyses are input data relating to Figure 4A. Mr indicates Markers’ molecular size. B) Bar graph depicts mean ± SEM proportion of deformed NMuMG organoids expressed as a percentage of total colonies scored for each experimental condition from three biological replicates including the one with representative differential interference contrast (DIC) images shown in 4C. C) Representative fluorescence microscopy scans of Actin-(TRITC-phalloidin-orange) stained fixed 8-day old organoids of NMuMG cells transfected and assessed as in 4B-D. Scale bars indicate 50 μm. D) Bar graph depicts mean ± SEM proportion of cortical actin organization expressed as a percentage of total colonies scored for each experimental condition from three biological replicates including the one with representative IF images shown in C. Statistical difference, ANOVA: *P≤0.05, **P≤0.01, ***P≤0.001.

**Figure S5: HDAC1 acts in a deacetylase-dependent manner to suppress EMT induction by endogenous/basal TGFβ signaling in mammary epithelial organoids.**

A) HDAC1 and actin (loading control) immunoblotting (IB) of lysates of NMuMG cells transiently transfected with vector controls, or one encoding the HDAC1-targetting shRNA (HDAC1i), or a plasmid encoding a deacetylase inactive HDAC (HDAC1YH). Mr indicates Markers’ molecular size. B) Bar graph depicts mean ± SEM proportion of HDAC1 immunoreactive band in each treatment condition from three independent experiments including the one shown in A. C) Representative differential interference contrast (DIC) light microscopy micrographs of live untreated or 10 µM KI-treated 8-day old organoids of NMuMG cells transfected and assessed as in A. Scale bar indicates 50 μm. Green and red arrows indicate hollow and filled/disorganized acinar organoids, respectively. D) Bar graph depicts mean ± SEM proportion of hollow NMuMG acinar organoids expressed as a percentage of total colonies scored for each experimental condition from three biological replicates including the replicate with representative differential interference contrast (DIC) images shown in C. E) Bar graph depicts mean ± SEM proportion of deformed NMuMG organoids expressed as a percentage of total colonies scored for each experimental condition from three biological replicates including the one with representative DIC images shown in C. Statistical difference, ANOVA: *P≤0.05, **P≤0.01, ***P≤0.001.

**Figure S6: Related to Figure 5.**

A) p300 (HA), SnoN and actin (loading control) immunoblotting (IB) of 20% of lysates of cells transiently transfected with a plasmid encoding only the Renilla luciferase protein (RLuc) (-), or in fusion with wild type SnoN (RLuc/SnoN) (+), the SUMO loss of function SnoNKdR (RLuc/SnoNKdR) (+), or the SUMO gain of function SUMO-SnoN (RLuc/SUMO-SnoN) (+), together with a vector control (-), or a plasmid encoding p300/HA. The immunoblotting analyses are input data relating to Figure 5A. B) Bar graph depicts mean ± SEM proportion of deformed NMuMG organoids expressed as a percentage of total colonies scored for each experimental condition from three biological replicates including the one with representative DIC images shown in 5C. C) Representative fluorescence microscopy scans of Actin-(TRITC-phalloidin-orange) stained fixed 8-day old organoids of NMuMG cells transfected and assessed as in 5B-D. Scale bar indicates 50 μm. D) Bar graph depicts mean ± SEM proportion of cortical actin organization expressed as a percentage of total colonies scored for each experimental condition from three biological replicates including the one with representative IF images shown in C. E) p300 (HA) and actin (loading control) immunoblotting (IB) of lysates of NMuMG cells transiently transfected with a vector control, or a plasmid encoding p300/HA. F) Representative differential interference contrast (DIC) light microscopy micrographs of live untreated or 10 µM KI-treated 8-day old organoids of NMuMG cells transfected and assessed as in E. Green and red arrows indicate hollow and filled/disorganized acinar organoids, respectively. G) Bar graph depicts mean ± SEM proportion of hollow NMuMG acinar organoids expressed as a percentage of total colonies scored for each experimental condition from three biological replicates including the replicate with representative differential interference contrast (DIC) images shown in F. H) Bar graph depicts mean ± SEM proportion of deformed NMuMG organoids expressed as a percentage of total colonies scored for each experimental condition from three biological replicates including the one with representative DIC images shown in F. Mr indicates Markers’ molecular size. Scale bars indicate 50 μm. Statistical difference, ANOVA: *P≤0.05, **P≤0.01, ***P≤0.001.

**Figure S7: Sumoylated SnoN acts via HDAC1 to suppress EMT in breast carcinoma organoids.**

A) SnoN, HDAC1 and actin (loading control) immunoblotting (IB) of lysates of MDA-MB-231 cells transfected with a stable vector control (-), or a plasmid stably expressing the wild type SnoN (WT), the SUMO loss of function SnoNKdR (KdR), or the SUMO gain of function SUMO-SnoN (SUMO), with each transiently transfected with vector controls (-), or a plasmid encoding the protein HDAC1 (+), or the HDAC1-targetting shRNA HDAC1i (+). B) Representative differential interference contrast (DIC) light microscopy micrographs of untreated (-) or 100pM TGFβ-treated (+) 8-day old organoids of MDA-MB-231 cells transfected and assessed as in A. Green and red arrows indicate non-deformed and deformed organoids, respectively. C) Bar graph depicts mean ± SEM proportion of non-deformed organoids expressed as a percentage of total colonies counted for each experimental condition from three biological replicates including the replicate with representative DIC images shown in B. Mr indicates Markers’ molecular size. Statistical difference, ANOVA: *P≤0.05, **P≤0.01, ***P≤0.001. Scale bar indicates 50 μm.

**Figure S8: Related to Figure 7.**

A) Representative fluorescence microscopy scans of Actin-(TRITC-phalloidin-orange) stained fixed 8-day old organoids of MDA-MB-231 cells transfected and assessed as in 7A-C. Scale bars indicate 50 μm. B) Bar graph depicts mean ± SEM proportion of cortical actin organization expressed as a percentage of total colonies scored for each experimental condition from three biological replicates including the one with representative IF images shown in A. *C-F) HDAC1 acts in a deacetylase-dependent manner to suppress endogenous/basal TGFβ-induced EMT in MDA-MB-231 organoids.* C) HDAC1 and actin (loading control) immunoblotting (IB) of lysates of MDA-MB-231 cells transiently transfected with vector controls, or one encoding the HDAC1-targetting shRNA HDAC1i, or a plasmid encoding the deacetylase inactive HDAC1YH. Mr indicates Markers’ molecular size. D) Bar graph depicts mean ± SEM proportion of HDAC1 immunoreactive band in each treatment condition from three independent experiments including the one shown in C. E-) Representative differential interference contrast (DIC) light microscopy micrographs of live untreated or 10 µM KI-treated 8-day old organoids of MDA-MB-231 cells transfected and assessed as in C. Green and red arrows indicate non-deformed and deformed organoids, respectively. F) Bar graph depicts mean ± SEM proportion of non-deformed organoids expressed as a percentage of total colonies scored for each experimental condition from three biological replicates including the replicate with representative DIC images shown in E. Statistical difference, ANOVA: *P≤0.05, **P≤0.01, ***P≤0.001. Scale bars indicate 50 μm.

**Figure S9: SnoN-HDAC1 complex acts in a sumoylation and deacytelase-dependent manner to suppress TGFβ-induced breast carcinoma cell migration.**

A) Representative differential interference contrast (DIC) light microscopy images taken at 0h and 30h following introduction of a scratch in overnight-serum-starved transfected MDA-MB-231 cell monolayers, and incubation without (-) or with (+) 100 pM TGFβ under serum starvation. For these experiments, MDA-MB-231 stable cells expressing SnoNWT, SnoNKdR, or SUMO-SnoN, or expressing the resistance marker only (vector control), were transiently transfected with a plasmid expressing HDAC1/FLAG, HDAC1YH/FLAG, or HDAC1-targetting shRNA HDAC1i, or with a mammalian expression control vector and/or RNAi control vector. Scale bar represents 500μm. The margins/boundaries of each scratch are highlighted with white dotted lines (-----) in each DIC micrograph. B) Bar graph depicts mean ± SEM proportion of scratch closure (%) at 30h with relative to the 0h of 3 non-overlapping images of each experimental condition from three independent biological replicated experiments including the one shown in A. Statistical difference, ANOVA: *P≤0.05, **P≤0.01, ***P≤0.001.

**Figure S10:** **Expression of** **p300 promotes endogenous/basal TGFβ-induced EMT in breast cancer organoids.**

A) p300 (HA) and actin (loading control) immunoblotting (IB) of lysates of MDA-MB-231 cells transfected transiently with a vector control, or a plasmid encoding p300/HA. Mr indicates Markers’ molecular size. B) Representative differential interference contrast (DIC) light microscopy micrographs of live untreated or 10 µM KI-treated 8-day old organoids of MDA-MB-231 cells transfected and assessed as in A. Green and red arrows indicate non-deformed and deformed organoids, respectively. Scale bar indicates 50 μm. C) Bar graph depicts mean ± SEM proportion of non-deformed MDA-MB-231 organoids expressed as a percentage of total colonies scored for each experimental condition from three biological replicates including the replicate with representative DIC images shown in B. Statistical difference, ANOVA: ***P≤0.001.

**Figure S11:** **SnoN-p300 complex acts in a sumoylation-dependent manner to suppress TGFβ-induced breast carcinoma cell migration.**

A) Representative differential interference contrast (DIC) light microscopy images taken at 0h and 30h following introduction of a scratch in overnight-serum-starved transfected MDA-MB-231 cell monolayers, and incubation without (-) or with (+) 100 pM TGFβ under serum starvation. For these experiments, MDA-MB-231 stable cells expressing SnoNWT, SnoNKdR, or SUMO-SnoN, or expressing the resistance marker only (vector control), were transiently transfected with a plasmid expressing p300/HA, or the p300-targetting shRNA p300i or with a mammalian expression control vector and/or RNAi control vector. Scale bar represents 500μm. The margins/boundaries of each scratch are highlighted with white dotted lines (-----) in each DIC micrograph. B) Bar graph depicts mean ± SEM proportion of scratch closure (%) at 30h with relative to the 0h of 3 non-overlapping images of each experimental condition from three independent biological replicated experiments including the one shown in A. Statistical difference, ANOVA: *P≤0.05, **P≤0.01, ***P≤0.001.
